# Epigenetically-controlled CEBPB regulates kidney cancer tumorigenesis via GPD1L-mediated ether lipid synthesis

**DOI:** 10.1038/s41419-025-08403-4

**Published:** 2026-01-22

**Authors:** Thi Ha Nguyen, Xuan Linh Mai, Tin Tin Manh Nguyen, Hoonsik Nam, Sunghyouk Park, Ji Yun Lee

**Affiliations:** Natural Products Research Institute, College of Pharmacy, Seoul National University, Seoul, Republic of Korea

**Keywords:** Cancer metabolism, Tumour biomarkers

## Abstract

Clear cell renal cell carcinoma (ccRCC) is characterized by disrupted lipid metabolism, traditionally attributed to *VHL* mutations and HIF stabilization. Here, we identified CEBPB as an epigenetically upregulated, VHL-independent transcription factor driving ccRCC tumorigenesis. CEBPB was regulated by H3K27ac and H3K4me and transcriptionally repressed the tumor-suppressive glycerol-3-phosphate dehydrogenase 1-like protein (GPD1L), thereby elevating dihydroxyacetone phosphate (DHAP)-derived ether lipid synthesis and enhancing Akt signaling. This activation suppressed CPT1A expression, inhibiting fatty acid oxidation (FAO) and leading to lipid accumulation, as found by lipidomics and isotope tracing. Loss of CEBPB reduced ether lipids, reactivated CPT1A, and impaired Akt signaling, diminishing tumor growth and lipid content in vitro and in vivo. Restoration of ether lipids or Akt activity rescued these effects. Importantly, CEBPB expression and enhancer activation were not modulated by VHL status and it could be targeted pharmacologically. The CEBPB-GPD1L-ether lipid-Akt-CPT1A axis is proposed as a new druggable driver in ccRCC integrating epigenetics, transcription, intermediary metabolism and oncogenic signaling.

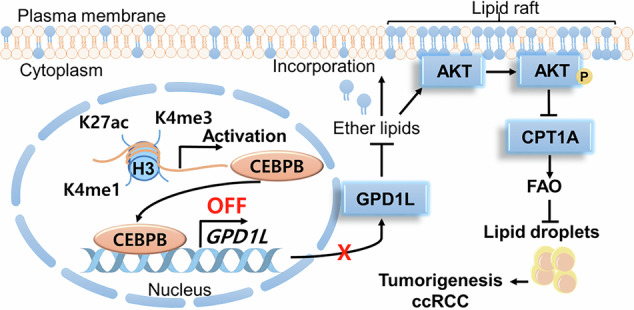

## Introduction

Reprogramming cellular metabolism is a critical hallmark of cancer, particularly in clear cell renal cell carcinoma (ccRCC), representing approximately 75% of kidney cancers [1, 2]. A key metabolic phenotype of ccRCC is the accumulation of cytoplasmic lipid droplets (LDs) [3, 4]. As *VHL* mutations, found in 50-80% of ccRCC, stabilize the transcription factor (TF) hypoxia-inducible factors (HIFs), this genotype-TF axis has been implicated in the characteristic lipid phenotype through several mechanisms, including fatty acid uptake by CD36, lysophosphatidylcholine acyltransferase 1/ ATP citrate lyase-mediated fatty acid production, activation of sterol regulatory element binding transcription factor (SREBP)-dependent fatty acid synthesis, and apolipoprotein L1- or Perilipin 2-dependent lipid storage [3, 5–9]. HIFs have been reported to suppress fatty acid oxidation (FAO) by downregulating enzymes like CPT1A and malonyl-CoA decarboxylase [10, 11]. However, recent research suggests that HIF stabilization alone may not fully explain LD accumulation, as *VHL-*mutant ccRCC cells with constitutive HIF stabilization lost the capacity for LD accumulation without concurrent MYC inhibition [12]. Furthermore, other pathways, such as jumonji domain-containing 6 (JMJD6)-diacylglycerol O-acyltransferase 1 (DGAT1) [13] and glutathione peroxidase 8 (GPX8)-nicotinamide N-methyltransferase [14], have been identified as contributing to lipid reprogramming, independently of VHL-HIF signaling. These data suggest that lipid accumulation in ccRCC may involve TFs or pathways beyond the traditional VHL-HIF framework.

CEBPB is a nuclear TF [15] that regulates cellular proliferation, differentiation [16–18], inflammation [19, 20], and metabolism [21, 22]. As a central TF for adipogenesis in adipocytes [23, 24], CEBPB likewise drives hepatic lipid metabolism in either SREBP1c-dependent or -independent manner, according to different mouse models [25–27]. CEBPB depletion decreased hepatic triacylglycerol (TG) by attenuating enzymes involved in lipogenesis and TG deposition [27]. *CEBPB* has been proposed as a pro-tumorigenic factor in various cancers, including breast cancer [28], esophageal squamous cell carcinoma [29], glioblastoma [30, 31], and acute lymphoblastic leukemia [32]. High expression of CEBPB was related to poor prognosis in nasopharyngeal carcinoma and gastric cancer via transcriptionally activating CPT1 and facilitating FAO [33, 34]. CEBPB has also been associated with ccRCC [35–37], but its detailed mechanism in lipid accumulation remains elusive.

GPD1L, a relatively understudied homologue of GPD1, along with GPD2, constitutes the glycerol 3-phosphate shuttle (GPS), which maintains cellular energy and redox balance [38]. GPD1L and GPD1 produce glycerol 3-phosphate (G3P) by reducing dihydroxyacetone phosphate (DHAP), a key glycolytic intermediate and rate-limiting precursor of ether lipids, thereby linking glucose and ether lipid metabolism. Ether lipids, due to their unique structure, can modulate membrane fluidity, stability, and lipid raft dynamics, influencing cellular signaling pathways like PI3K/Akt, ERK/MAPK, and NF-κB [39–42]. Although understudied, ether lipids are reported to accumulate in cancer, promoting the above oncogenic signaling [42, 43]. GPD1L has been reported as a tumor suppressor in several cancers, including colorectal cancer via HIF-1α stabilization and matrix metalloproteinase-9 transcription [44] and esophageal squamous cell carcinoma via PI3K/Akt signaling pathway [45]. Similarly, GPD1L suppressed ccRCC through PINK1/Parkin-mediated mitophagy [46] or CPT1A-regulated FAO [47]. However, a recent study suggested GPD1L as oncogenic, supplying G3P for phospholipid synthesis in ccRCC, contradicting the earlier reports on GPD1L and ether lipids in cancer [48]. Its role thus remains controversial.

Given that the VHL-HIF axis may not be the only TF involved in lipid accumulation in ccRCC, we tried to find an alternative TF directly involved in both lipid accumulation and tumor progression.

## Materials and methods

For the detailed experimental procedures, please refer to the Supplementary Methods (available online).

### Cell culture

All cells were cultured in DMEM supplemented with 10% fetal bovine serum and 1% penicillin-streptomycin.

### CEBPB KD

Lentivirus was produced by transfecting HEK293T cells with lentiviral vectors expressing shCEBPB or shCTR, PLP-1, PLP-2, and VSV-G, and the viral solution was transduced to target cells.

### Xenograft mouse

Xenograft of shCTR, shCEBPB#1, and shCEBPB#2 Caki-1 cells were performed to BALB/c nude mice (male, 6 weeks old) as previously reported [14]. The whole procedure was approved by Seoul National University IACUC (Number: SNU-240417-1).

### Lipid droplet staining and Luciferase reporter assay

Standard procedures were used with Oil Red O, BODIPY 493/503, Nile red, and pEXZ-PG04-*GPD1L* promoter reporter plasmid.

### Chromatin immunoprecipitation (ChIP)-PCR

CEBPB antibody and anti-rabbit IgG were used as an IP antibody and negative control [49].

### LC/MS/MS untargeted lipidomics

LC/MS/MS was performed as previously reported [14, 41].

### Statistical analysis

The un-paired *t*-test was used to analyze two sets of data, while two-way ANOVA with Geisser-Greenhouse correction and a post hoc Dunnett-*t*-test were applied for multiple comparisons. The Mann–Whitney U test was used in TCGA data analysis.

## Results

### CEBPB is an epigenetically-regulated TF associated with higher grade and poor prognosis in ccRCC

**Fig. 1:**
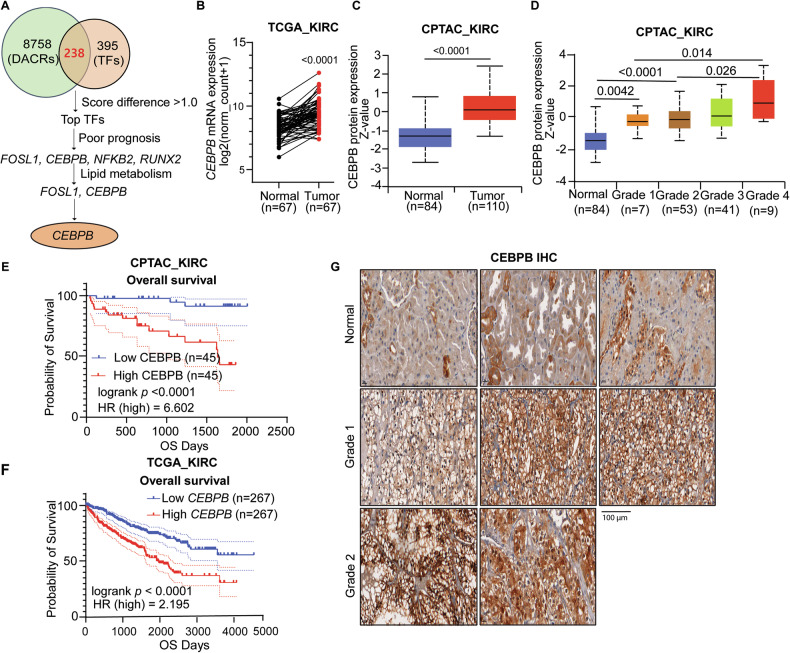
CEBPB is an epigenetically-regulated TF associated with higher grade and poor prognosis in ccRCC. **A** Bioinformatic screening of evaluating new TFs related to lipid metabolism in ccRCC. The data for ccRCC-specific DACRs and TFs were extracted from the work of Terekhanova et al. [50]. The TF candidates should meet all the following criteria: (1) score difference of TF between ccRCC and proximal tubule cells should be above 1; (2) its expression should be higher in ccRCC tumors than in normal tissues in TCGA-KIRC database; (3) its higher expression should be related to a worse ccRCC OS; and (4) it should be correlated with lipid metabolism in ccRCC tissue in the TCGA dataset. **B** *CEBPB* mRNA level in normal and paired tumors (n = 67) (TCGA-KIRC cohort). **C**, **D** CEBPB protein level in normal and ccRCC tumor tissues (**C**), according to neoplasm histologic grade (**D**) in CPTAC-KIRC dataset (https://ualcan.path.uab.edu). **E**, **F** Kaplan–Meier analysis of overall survival for ccRCC patients based on CEBPB protein level (**E**) and mRNA expression (**F**) (cutoff: median). The mRNA expression, protein levels and clinical characteristics of ccRCC patients were collected from TCGA-KIRC and CPTAC-KIRC. **G** CEBPB IHC staining for TMA (Cat# KD485, Biomax) from ccRCC patients. Magnification 400×. Data presented in (**C**, **D**) are means ± SD (n > 3). *p*-value was calculated by Mann–Whitney U test for (**B**), two-sided *t*-test for (**C**, **D**), log-rank (Mantel-Cox) test for (**E**, **F**).

We tried to find a TF related to lipid metabolism in ccRCC, focusing on TFs regulated by epigenetic changes rather than *VHL* mutations. We exploited 15,768 cancer-cell-specific differentially accessible chromatin regions (DACRs) that represent 8996 genes based on snATAC-seq comparing ccRCC and proximal tubule cells (PTCs) [50]. To narrow our focus to TFs, we intersected these genes with 633 TFs having increased motif accessibility in cancer cells compared to PTCs, hence, enhanced TF activity. This yielded 238 TFs, which have increased accessibility and enhanced expression/activity in ccRCC. Subsequent filtering with various criteria, including lipid metabolism, gave two TFs, FOS Like 1 (FOSL1) and CEBPB (Figs. 1A and S1A, B; Table S1, see the Fig. 1A legend for details).

Both genes were negatively correlated with the fatty acid oxidation/catabolism in ccRCC (Table S1). While FOSL1 is a TF regulating cell differentiation/proliferation, CEBPB is a key TF regulating adipogenesis in adipocytes. As ccRCC cells exhibit adipocyte-like properties [9], we decided to further explore CEBPB. As CEBPB was identified through accessibility screening, we further sought specific epigenetic modifications at its regulatory sites. Upon querying the ChIP-seq profiles of three epigenetic marks (H3K27ac, H3K4me1, and H3K4me3) in 10 ccRCC primary tumor/normal pairs (GSE86095) [51], we identified gained H3K27ac and enrichment of H3K4me1 and H3K4me3 at distal enhancers of CEBPB in ccRCC tumors (Figs. S1C, D). Furthermore, gained H3K27ac signals at a “super-enhancer” near CEBPB were observed in ccRCC tumors (Fig. S1E). These epigenetic changes turned out to be strongly correlated with the significant overexpression of CEBPB in tumors compared to that in normal samples [51]. Consistent enhancer activation patterns were found in 786-O and A498 cells compared to a normal renal proximal cell line (HKC8) (Fig. S1F), suggesting histone modifications drive CEBPB upregulation in ccRCC.

We then evaluated the clinical relevance of CEBPB in ccRCC based on mRNA (TCGA) and protein expression (CPTAC). *CEBPB* mRNA and protein level were higher in tumors than in normal tissues (Figs. S2A and 1B, C). Notably, CEBPB protein level increased with ccRCC grades (Fig. 1D), consistent with the mRNA expression results (Fig. S2B). As for prognosis, higher protein level of CEBPB was associated with lower overall survival (OS) of the ccRCC patients (Fig. 1E). High *CEBPB* expression was correlated with lower OS and progression-free interval (PFI) in ccRCC patients (Figs. 1F and S2C), but not in KICH (chromophobe kidney cancer) and KIRP (kidney renal papillary carcinoma) patients (Fig. S2D), indicating ccRCC-specific CEBPB prognosis relationship. Higher expression of *CEBPB* was also positively associated with metastasis, deaths from tumors, and progression after primary therapies (Figs. S2E–G), further confirming CEBPB’s strong association with ccRCC prognosis.

In addition to the public cohort analysis, our own immunohistochemical (IHC) staining of ccRCC patient tissues confirmed higher CEBPB protein levels in cancer tissues compared to normal tissues (Fig. 1G). Taken together, the epigenetically regulated CEBPB is associated with both tumorigenesis and worse prognosis in ccRCC.

### CEBPB promotes ccRCC in vitro and in vivo

**Fig. 2:**
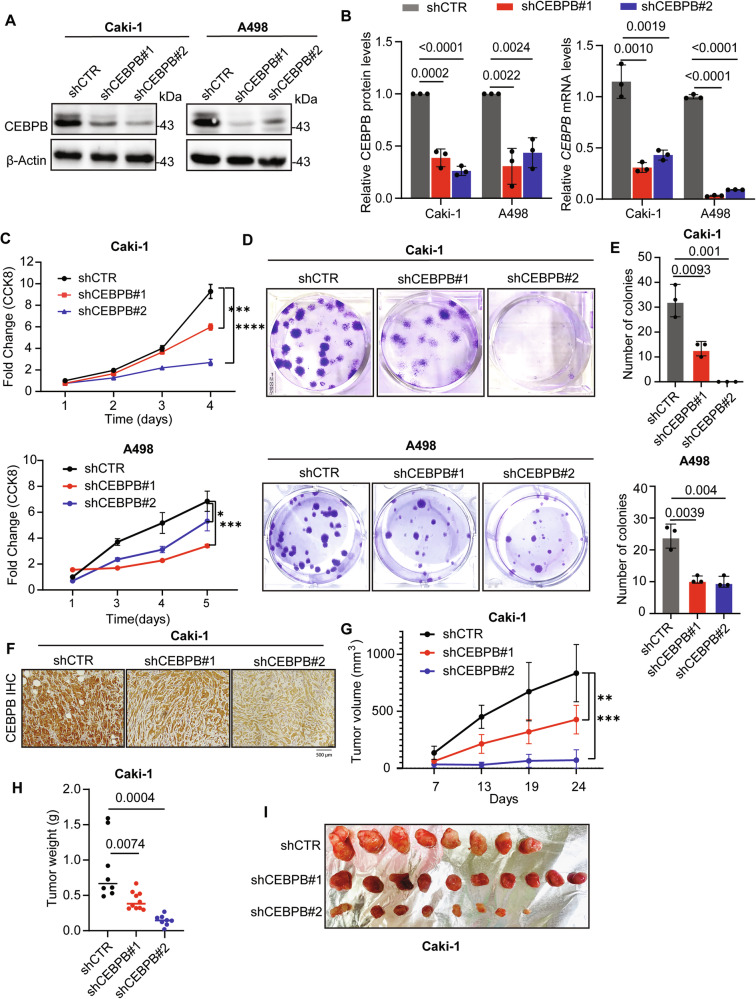
CEBPB promotes ccRCC in vitro and in vivo. **A**-**E** Representative immunoblot of CEBPB (**A**), relative CEBPB protein levels normalized to β-Actin protein levels and qPCR analysis of *CEBPB* mRNA expression normalized to *GAPDH* (n = 3) (**B**), relative growth rates measured by CCK8 kit (n = 3) (**C**), and colony formation and corresponding quantification data (n = 3) (**D**, **E**) in Caki-1 and A498 cell lines transduced with shCTR, shCEBPB#1, and shCEBPB#2. **F**-**I** Xenograft tumor in nude mice (n ≥ 8) after inoculating Caki-1 shCTR, shCEBPB#1, and shCEBPB#2 transfected cells. CEBPB IHC staining of tumor tissue (**F**), tumor volume after 7, 13, 19, 24 days of inoculation (**G**), tumor weight after dissection (**H**), and images of tumor after dissection (**I**). Error bars represent mean ± SD (n ≥ 3), *p*-value was determined by un-paired *t*-test for panels (**B**, **E**, **H**); two-way ANOVA with Geisser-Greenhouse correction for (**C**, **G**). ^*^p < 0.05, ^**^p < 0.01,^***^p < 0.001, ^****^p < 0.0001.

To establish the causal involvement of CEBPB in ccRCC tumorigenesis, we silenced its expression both transiently using siRNA and stably using shRNA in two ccRCC cell lines (Caki-1 and A498) (Figs. S2H and 2A, B). Genetic knockdown (KD) by the two shRNAs decreased cell proliferation and colony formation in both cell lines compared with the control group (Figs. 2C–E). Consistent results were obtained using siRNA (Figs. S2I–K). To examine the tumorigenic capacity of CEBPB, we performed xenografts in nude mice with shCEBPB-transfected Caki-1 cells. CEBPB KD effectively depleted CEBPB in the tissue (Fig. 2F) and decreased tumor volume, weight, and size (Figs. 2G–I). For all in vivo phenotypes with Caki-1, shCEBPB#2, with higher suppression of CEBPB expression than shCEBPB#1, exhibited a larger effect, indicating that CEBPB plays a causal role in ccRCC tumorigenesis.

### CEBPB is important in altered lipid metabolism in ccRCC

**Fig. 3:**
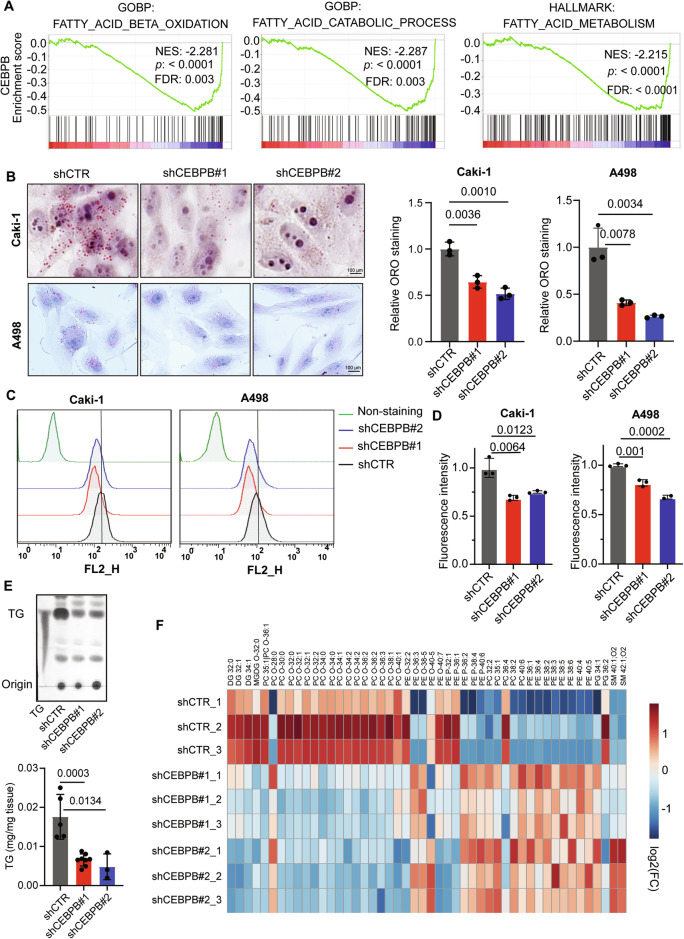
CEBPB is important in altered lipid metabolism in ccRCC. **A** GSEA of the mRNA expression of *CEBPB* in TCGA-KIRC database and FAO, fatty acid catabolic processing, and fatty acid metabolism. FDR < 0.05 and *p* < 0.05 were considered significant. **B** Representative images (left) of ORO staining for detecting lipid content in Caki-1 and A498 shCEBPB. Quantitation of ORO staining (right) (n = 3). **C** Caki-1 and A498 shCEBPB cells were stained with 5 μM Nile red in PBS for 20 min at 37 °C, and red fluorescence intensity of labelled lipid measured by FACS with FL2 channel. **D** Quantitation of the fluorescence intensity of Nile red labelled lipid in (**C**) (n = 3). **E** TLC analysis of TG (top) and quantification of TG amount normalized to tissue weight using Chekine^TM^ Micro TG assay kit (bottom, n ≥ 3) in in vivo xenograft tumor tissues of nude mice with shCTR and shCEBPB Caki-1 cell transplantation. **F** Untargeted lipidomics: Changes in lipid profile upon CEBPB KD. Metabolites with a |fold change| > 1.2, and *p*-value < 0.05, were considered to be significantly changed. Heat-map of significantly different lipid species levels from untargeted lipidomics comparing shCEBPB vs. shCTR Caki-1 (n = 3). Error bars represent mean ± SD (n = 3). *p*-value was calculated by un-paired *t*-test for panels (**D**).

To explore the roles of CEBPB in ccRCC tumorigenesis, we analyzed gene expression correlations from TCGA-KIRC and performed GSEA using Gene Ontology (GO) and Hallmarks gene sets. Most of the top 20 negatively correlated pathways were metabolic, with high enrichment of lipid metabolism-related gene sets (Figs. S3A, B). Notably, fatty acid metabolism and FAO exhibited strong negative enrichment (Figs. 3A and S3C). As lower FAO can lead to lipid accumulation [10], we hypothesized that CEBPB may promote lipid accumulation in ccRCC by suppressing FAO.

To test this, we measured LD levels upon CEBPB KD in Caki-1 and A498 cells. CEBPB depletion significantly decreased the size and number of LDs, as shown by Oil Red O (ORO) staining (Fig. 3B), BODIPY 493/503 staining (Fig. S3D), and Nile red fluorescence (Figs. 3C, D). These in vitro results were validated in an in vivo xenograft model, where TG levels were lower in the tumors from the shCEBPB Caki-1-bearing mice compared with the shCTR-xenografted ones (Fig. 3E). Therefore, CEBPB enhanced overall neutral lipid accumulation in ccRCC.

As TG and ORO-stained lipids are storage lipids, we further investigated other CEBPB-modulated lipid species bearing possible mechanistic implications. To that end, we performed a lipidomic analysis on shCEBPB and shCTR Caki-1 cells (Figs. S3E, F). CEBPB KD decreased the level of another neutral lipid species, diacylglycerol (DG), reflecting depleted LDs (Fig. 3F). Interestingly, ether phospholipids (plasmanyl ether of phosphatidylcholine (PC) and phosphatidylethanolamine (PE)) were also significantly downregulated, whereas glycerophospholipids (PC, PE) were elevated. These results suggest that CEBPB differentially regulates neutral lipids and ether lipids, which may have mechanistic implications.

### CEBPB regulates ether lipid synthesis via GPD1L modulation

**Fig. 4:**
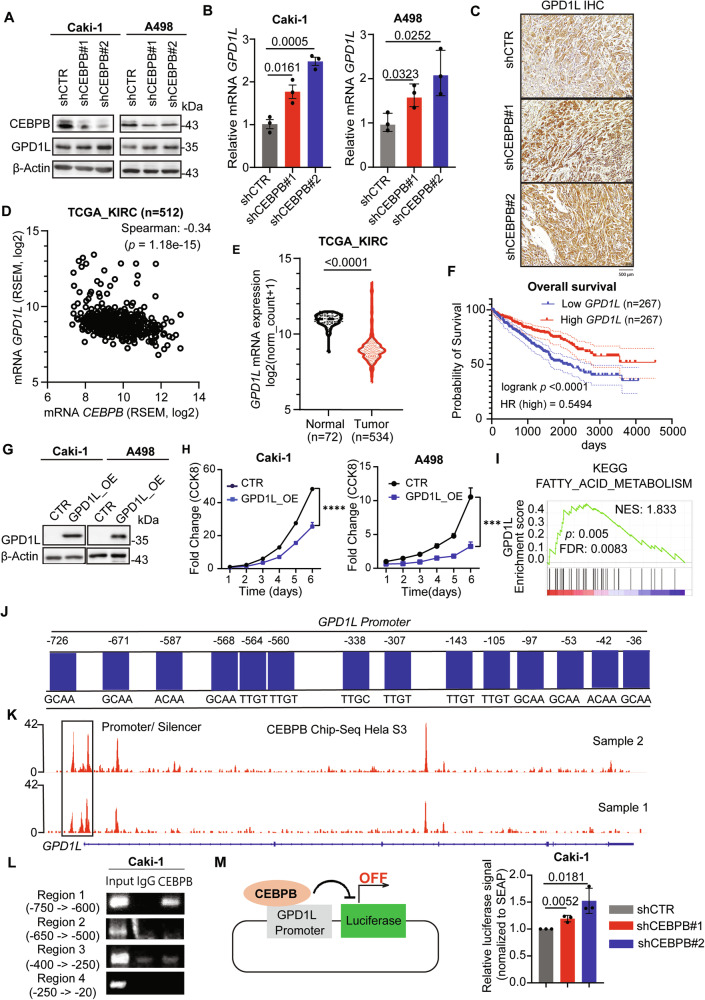
CEBPB regulates ether lipid synthesis via GPD1L modulation. **A**, **B** Protein levels (**A**) and mRNA expression (**B**) of GPD1L in Caki-1 and A498 cells with shCEBPB and shCTR (n = 3). **C** Presentative images of GPD1L IHC staining of tumors from in vivo xenografted mice with Caki-1 shCEBPB and shCTR. **D** Correlation between *CEBPB* and *GPD1L* mRNA expression obtained from TCGA-KIRC database. **E** Violin plots for the mRNA expression levels of *GPD1L* in normal and tumor tissue from ccRCC patients (TCGA-KIRC). **F** Kaplan-Meier analysis of *GPD1L* mRNA expression patterns on OS in ccRCC patients (cutoff: median). **G**, **H** The mRNA expression values and clinical characteristics of ccRCC patients were collected from TCGA-KIRC database. Immunoblotting analysis of GPD1L (**G**) and relative growth rates (n = 3) (**H**) in Caki-1 and A498 with GPD1L or scramble OE. **I** GSEA of the mRNA expression of *GPD1L* in TCGA-KIRC database and lipid metabolism. **J** CEBPB binding sites in the *GPD1L* promoter are predicted in PROMO (ALGGEN) version V.8.3. of TRANSFAC (five maximum matrix dissimilarity rate). **K** Analysis CEBPB ChIP-seq data in Hela-S3 cells with peaks in *GPD1L* regions by employing Integrative Genomic Viewer (IGV) (https://igv.org/app/). **L** ChIP-PCR assay of CEBPB binding regions on *GPD1L* promoter in Caki-1 cells. **M** Luciferase activity assay of GPD1L in Caki-1 cells transfected with shCTR or shCEBPB (sh#1, sh#2) (n = 3). Error bars are means ± SD (n = 3). *p*-value was obtained by un-paired *t*-test for (**B**, **M**); Mann–Whitney U test for (**E**), log-rank (Mantel-Cox) test for (**F**), two-way ANOVA with Geisser-Greenhouse correction for (**H**). ^***^p < 0.001, ^****^p < 0.0001.

Among the changes in lipid classes upon CEBPB KD, we focused on ether lipid metabolism, as ether lipid synthesis, mediated by GPD2, has been implicated in various cancers [41] and ether lipid levels were elevated in ccRCC patient tissues [52]. These led us to investigate the relationship between CEBPB and members of GPD family (GPD1, GPD2, and GPD1L), which can all affect ether lipid levels by modulating the DHAP/G3P ratio. CEBPB KD increased both protein and mRNA levels of GPD1L (Figs. 4A, B) without affecting GPD1 or GPD2 (Fig. S4A). This increase was also recapitulated in the in vivo xenograft model with CEBPB suppression (Fig. 4C). Additionally, *CEBPB* exhibited a strong negative correlation with *GPD1L* (Spearman’s correlation: -0.344, *p*-value: 1.18e-15) from the TCGA-KIRC database (Fig. 4D). Therefore, we further studied the functional involvement of GPD1L in ccRCC in relation to CEBPB.

GPD1L exhibited nearly opposite characteristics to CEBPB: much lower mRNA and protein expression in ccRCC tumors, particularly in higher grade and stage (Figs. 4E and S4B–E). Its level was associated with longer OS (Figs. 4F and S4F), which was not observed in other types of kidney cancer (Fig. S4G). Lower *GPD1L* expression was also associated with progressive metastatic status (Fig. S4H). These cohort-level tumor-suppressive data were consistent with our in vitro data, where transient GPD1L overexpression (OE) lowered ccRCC cell proliferation (Figs. 4G, H). *GPD1L* expression was also positively correlated with fatty acid metabolism (Fig. 4I), in contrast to CEBPB’s negative correlation. These findings suggest that GPD1L has the typical characteristics of a tumor suppressor, specifically in ccRCC, and antagonizes CEBPB’s roles in lipid metabolism.

As CEBPB is a well-known TF, we also investigated whether CEBPB directly regulates GPD1L transcription. PROMO analysis [53, 54] revealed multiple putative CEBPB-binding sites in the GPD1L promoter (Fig. 4J), supported by public ChIP-seq data (GSM935553) (Fig. 4K). In comparison, CEBPB didn’t occupy the regions around the GPD1 and GPD2 promoters (Fig. S4I), consistent with our expression results. Moreover, the CEBPB-occupied region proved to be a silencer of GPD1L (chr3:32106620-32106763), through which CEBPB could block GPD1L transcription, supporting the antagonistic relationship. Furthermore, ChIP-PCR confirmed CEBPB binding to region 1 (−750 bp to −600 bp) in the GPD1L promoter (Fig. 4L), and a dual-luciferase reporter assay showed that CEBPB KD increased GPD1L promoter activity (Fig. 4M). Overall, GPD1L, which can affect ether lipids, was transcriptionally repressed by CEBPB in ccRCC.

### CEBPB-GPD1L axis-mediated ether lipid synthesis regulates Akt activity

**Fig. 5:**
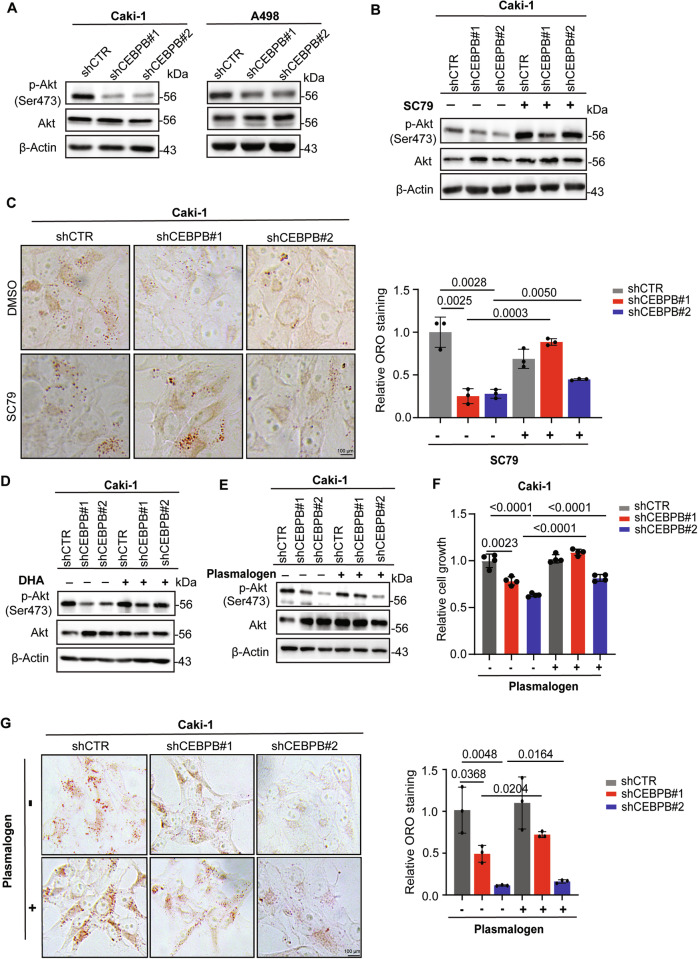
CEBPB-GPD1L axis-mediated ether lipid synthesis regulates Akt activity. **A** Expression of Akt and p-Akt (Ser473) in shCEBPB and shCTR Caki-1 and A498 cells, as detected by immunoblotting analysis. **B** Expression of Akt and p-Akt (Ser473) in shCEBPB and shCTR Caki-1 with DMSO or SC79 8 µg/mL treatment for 30 min, as detected by immunoblotting analysis. **C** Representative images (left) of ORO staining for detecting lipid content in shCEBPB and shCTR Caki-1 supplied with DMSO or SC79 100 ng/mL for 24 h. Quantitation of ORO staining of cells (right) (n = 3). **D** Expression of Akt and p-Akt (Ser473) in shCEBPB and shCTR Caki-1 cells and A498 cells with or without DHA 5 mM treatment for 2 h, as detected by immunoblotting analysis. **E** Expression of Akt and p-Akt (Ser473) in shCEBPB and shCTR Caki-1 cells with or without plasmalogen PC (18:0p/18:1) 200 µM treatment for 48 h, as detected by immunoblotting analysis. **F** Relative growth rate of shCEBPB and shCTR Caki-1 cells with or without plasmalogen PC (18:0p/18:1) 200 µM treatment for 72 h (n = 3). **G** Representative images of ORO staining for detecting lipid content in shCEBPB and shCTR Caki-1 supplied with or without plasmalogen PC (18:0p/18:1) 200 µM for 48 h. Quantitation of ORO staining of cells (right) (n = 3). Error bars are means ± SD (n = 3). *p*-value was obtained by un-paired *t*-test for (**C**, **F**, **G**).

With GPD1L repression and ether lipid elevation by CEBPB, we explored how these changes affect ccRCC tumorigenesis. Ether lipid enrichment in lipid rafts [39], Akt recruitment to lipid rafts [55], and the importance of Akt signaling in cancers [56, 57] have been reported. In addition, ether lipid synthesis depends on DHAP, the rate-limiting precursor, whose production is inhibited by GPD1 or GPD1L, which shifts the DHAP ↔ G3P balance toward G3P. Therefore, we hypothesized that CEBPB could modulate Akt activity via DHAP-driven ether lipid synthesis. CEBPB KD reduced Akt activity by decreasing Akt phosphorylation at Ser473 (Fig. 5A), while Akt activation by SC79 reversed this reduction (Fig. 5B) and rescued the lipid depletion in shCEBPB-transfected cells (Fig. 5C), placing Akt downstream of CEBPB.

To examine the relationship between Akt and DHAP-driven ether lipid synthesis, we treated shCEBPB ccRCC cells with either 1,3-dihydroacetone (DHA), a cell-permeable precursor of DHAP [41, 58], or plasmalogen PC ether lipid (18:0p/18:1). Both restored Akt signaling (Figs. 5D, E), indicating that CEBPB’s pro-tumorigenic function is mediated by the Akt regulated by DHAP-driven ether lipid synthesis. Significantly, the ether lipid supplementation also rescued the inhibition of cell growth (Fig. 5F) and lipid depletion in shCEBPB Caki-1 cells (Fig. 5G). Therefore, these results indicate that CEBPB affects lipid metabolism and proliferation of ccRCC through ether lipid-driven Akt signaling.

### CEBPB inhibits CPT1A expression via Akt activity

Akt signaling is well-known to affect LDs in cancers [59, 60], and we investigated how CEBPB affects lipid accumulation in ccRCC via Akt. Since FAO-related pathways showed a significant negative relationship with CEBPB (Figs. 3A and S3C), we assumed that CEBPB regulates lipid accumulation via FAO. Indeed, CEBPB KD in Caki-1 and A498 cells significantly increased ^13^C acetyl-CoA formed from the oxidation of U-^13^C_16_-palmitic acid (Fig. S5A), confirming that CEBPB affects lipid accumulation via FAO regulation.

**Fig. 6:**
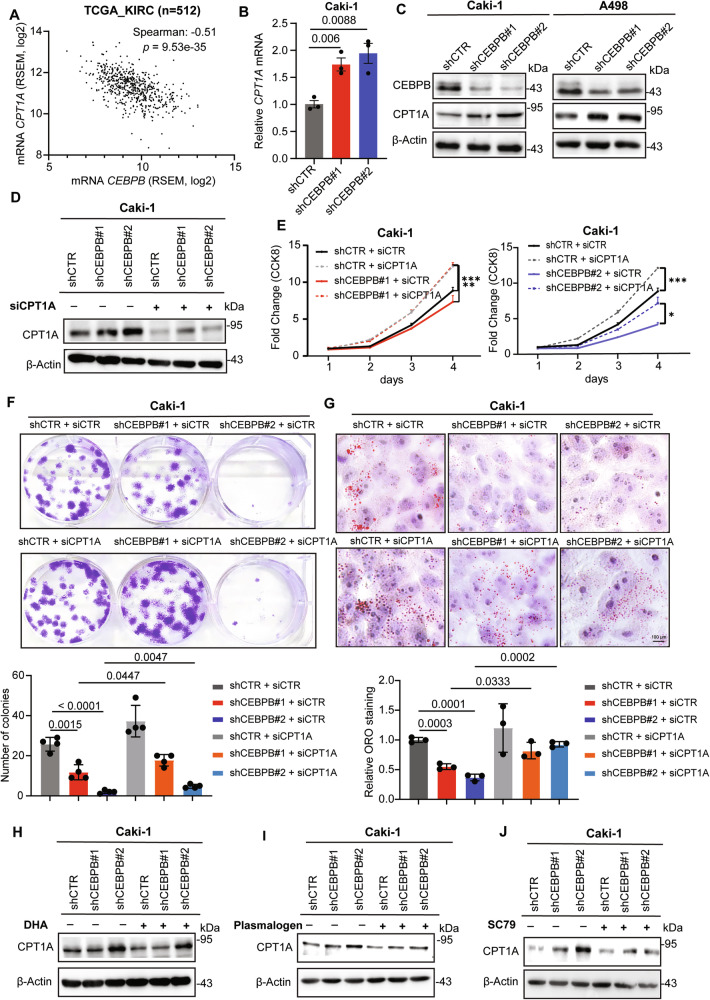
CEBPB inhibits CPT1A expression via Akt activity. **A** Negative correlation between *CEBPB* mRNA and *CPT1A* mRNA level from TCGA-KIRC database. **B**, **C** mRNA and protein levels of CPT1A in shCEBPB and shCTR Caki-1 and A498 cells. **D**-**G** Immunoblotting analysis of CPT1A, as normalized to β-Actin (**D**), cell growth rate (n = 3) (**E**), colony formation (top) and corresponding quantification data (n = 3) (bottom) (**F**), and representative images (top) and quantitation of ORO lipid staining (n = 3) (bottom) (**G**) of shCEBPB and shCTR Caki-1 cells upon CPT1A KD by siRNA CPT1A transfection (n = 3). **H**-**J** Immunoblotting analysis of CPT1A in Caki-1 shCEBPB upon 5 mM DHA treatment for 2 h (**H**), 200 µM plasmalogen PC (18:0p/18:1) treatment for 48 h (**I**), SC79 8 µg/mL treatment for 30 minutes (**J**). Error bars represent means ± SD (n = 3). *p*-values were obtained by un-paired *t*-test for (**B**) and two-way ANOVA with Geisser-Greenhouse correction for (**E**).

We further focused on CPT1A, a well-known rate-limiting enzyme in FAO, reportedly regulated by CEBPB in other cancers [33, 34]. This was supported by the strong negative correlation between *CEBPB* and *CPT1A* in TCGA ccRCC cohort (Fig. 6A) and by our RT-qPCR and immunoblotting results showing increased expression of CPT1A upon CEBPB KD (Figs. 6B, C). In vivo, IHC staining of xenografted tumors also showed induced CPT1A protein expression by CEBPB KD (Fig. S5B). Importantly, CPT1A KD in CEBPB-deficient cells recovered cell growth, colony formation, and lipid content (Figs. 6D–G), supporting CEBPB regulates LD formation and proliferation in ccRCC cells via CPT1A.

We also investigated how the GPD1L-ether lipid-Akt axis regulates CPT1A. Treatment with either DHA or plasmalogen PC (18:0p/18:1) decreased CPT1A expression, which was enhanced in CEBPB KD cells (Figs. 6H, I). Additionally, Akt activation by SC79 treatment reversed the CPT1A increase in shCEBPB cells (Fig. 6J). The relationship between Akt and CPT1A was also observed in non-transfected ccRCC cells, as SC79 treatment decreased CPT1A levels (Fig. S5C). To understand how Akt affects CPT1A expression, we evaluated ChIP-Seq data of well-known TFs modulated by Akt [56]. Among these, FOXO1, which is inhibited by Akt-mediated phosphorylation, turned out to bind to CPT1A enhancer regions (Fig. S5D). Therefore, Akt suppresses CPT1A by possibly inhibiting FOXO1, though other pathways cannot be excluded. Overall, our data suggest that CEBPB enhances lipid accumulation and proliferation in ccRCC by activating Akt, which suppresses CPT1A expression.

### CEBBP expression is VHL-independent

**Fig. 7:**
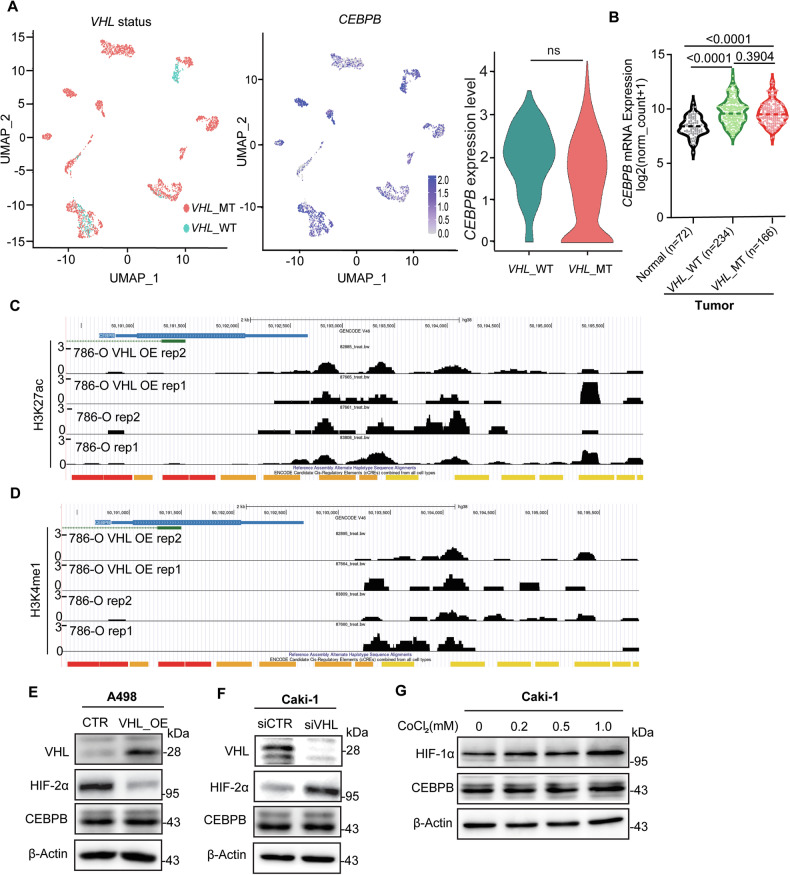
CEBPB expression is VHL-independent. **A** Single-cell atlas based on *VHL*-MT, *VHL*-WT status, CEBPB expression, and violin plot of CEBPB expression in cell clusters with or without *VHL* mutation from scRNA analysis (GSE159115). **B** mRNA expression levels of *CEBPB* according to normal and *VHL* status of tumors in ccRCC patients from TCGA-KIRC database. **C**, **D** Unaltered enhancer H3K27ac (GSM2723830, GSM2723822, GSM2723826, GSM2723818) (**C**) and H3K4me1 (GSM2723831, GSM2723823, GSM2723819, GSM2723827) (**D**) signals of CEBPB enhancer by VHL loss in 786-O cells. **E**, **F** Immunoblotting analysis of CEBPB, HIF-2α, and VHL, as normalized to β-Actin, upon VHL OE in A498 cells (**E**), VHL siRNA in Caki-1 cells (**F**). **G** Immunoblotting analysis of CEBPB and HIF-1α, as normalized to β-Actin, upon CoCl_2_ treatment in Caki-1 cells. *p*-value was obtained by *t*-test for (**A**), Mann–Whitney U test for (**B**).

*VHL* mutations and the resulting stabilization of HIF represent the most extensively studied pathway in ccRCC. Therefore, we examined whether *VHL* mutations regulate CEBPB. Re-analysis of published scRNA-seq data from ccRCC patients (GSE159115) showed no significant difference in CEBPB expression according to the *VHL* mutation status (Figs. S6A, B and 7A), which was confirmed in the TCGA-KIRC cohort (Fig. 7B). Beyond HIF activation, *VHL* loss can induce epigenetic changes in genes essential for ccRCC, particularly those with active H3K27ac signals in their enhancers and promoters [51]. However, ChIP-seq analysis (GSE102095) revealed no difference in H3K27ac and H3K4me1 profiles at CEBPB regulatory regions between VHL-deficient and VHL-restored 786-O cells (Figs. 7C, D). Similar results were obtained in A498 cells and patient-derived cell lines after VHL restoration (Figs. S6C–E). Confirming these, VHL OE in A498 cells or KD in Caki-1 cells did not alter CEBPB protein levels, whereas it modulated its well-known downstream target, HIF-2α (Figs. 7E, F). In addition, CoCl_2_ treatment in Caki-1 to enhance the VHL degradation and increase HIF-1α did not affect the CEBPB protein levels (Fig. 7G). Therefore, CEBPB is an important TF for ccRCC, and unlike HIF TF, its level is not regulated by *VHL* status.

### CEBBP as a valid pharmacological target for ccRCC

**Fig. 8:**
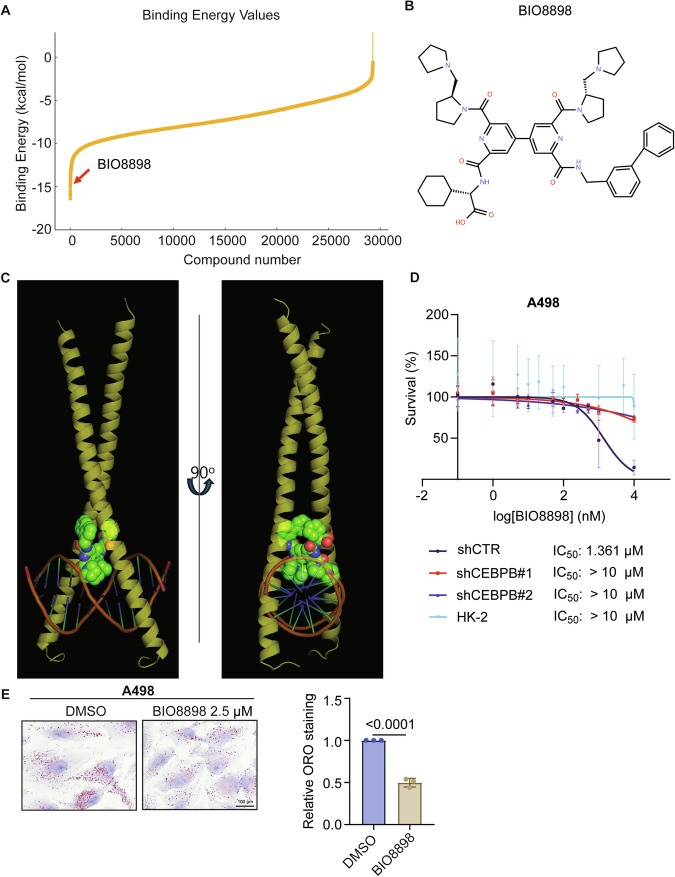
CEBBP as a valid pharmacological target for ccRCC. **A** Binding energy of 29,314 ligands with CEBPB-DNA complex from a structure-based virtual screening with BIO8898 among the top ten candidates with the lowest binding energy. **B** Structure of BIO8898. **C** 3D docking image of BIO8898 and CEBPB-DNA complex (PDB ID: 8K8D). **D** Effect of BIO8898 on the cell viability of A498 with shCTR, shCEBPB#1, shCEBPB#2 and HK-2 cells. Cell lines were treated with a range of concentration of BIO8898 (0.1 nM – 10 µM) for 72 h, and cell viability was determined by SRB assay. **E** Representative images (left) of ORO staining for detecting lipid content in A498 treated with DMSO or BIO8898 2.5 µM for 72 h. Quantitation of ORO staining (right) (n = 3). Error bars represent means ± SD (n = 3). *p*-values were obtained by un-paired *t*-test for (**E**).

Mechanistic studies on cancer, especially those involving TFs, often suggest targets that lacks druggability [61, 62]. As CEBPB is a leucine zipper-type TF with an empty space between its “zipper” domain and the DNA-binding region, we thought it could be exploited for pharmacological inhibition. Upon virtual screening of CEBPB-DNA complex structure (PDB ID: 8K8D) against 29,314 ligands, we identified BIO8898 as a candidate (Fig. 8A). It was originally developed to inhibit CD40-CD154 interaction [63] and exhibited a binding energy of −14.84 kcal/mol with extensive interaction with CEBPB-DNA complex (Figs. 8B, C). BIO8898 demonstrated strong inhibition on A498 cells (shCTR) with a single-digit micromolar IC_50_ (1.3 μM), significantly more effective than its original CD40-CD154 inhibition (IC_50_ of 25 μM). Notably, its effect was much weaker (IC_50_ > 10 μM) for CEBPB KD A498 cells (shCEBPB#1 or shCEBPP#2) or nearly absent for normal kidney cells (HK-2) (Fig. 8D). In addition, BIO8898 treatment to A498 cells significantly reduced the size and number of LDs as shown by ORO staining (Fig. 8E). These findings suggest that CEBPB is a viable target for small-molecule inhibitors, showing its translational implication.

## Discussion

ccRCC is characterized by disrupted lipid metabolism, commonly linked to *VHL* mutations. In this study, we identified CEBPB as a VHL-independent TF that is epigenetically regulated and crucial for lipid metabolism in ccRCC. We also suggest that CEBPB exerts its tumorigenic and lipogenic effects by regulating the GPD1L-ether lipids-Akt-CPT1A axis, demonstrating how tumorigenic TF signals can be transmitted to proteins via intermediary metabolites, such as ether lipids and DHAP.

Although CEBPB’s involvement in ccRCC has been previously reported [35, 64], its involvement and detailed mechanism in lipid metabolism have not been investigated. In the previous study, IL-6/STAT3 signaling was suggested to be downstream of CEBPB without detailed mechanistic insights. Our previous work revealed GPX8 activates IL-6/STAT3 pathway to drive de novo lipogenesis (DNL) [14]. However, in the current study, the CEBPB’s effects on DNL enzymes (ACC and FASN), TG/LD synthesis enzymes (DGAT1 and PLIN2), and DNL activity measured through CH_3_ ω-methyl ^13^C incorporation were inconsistent between Caki-1 and A498 cells (Figs. S7A–C). Moreover, CEBPB KD did not modulate GPX8 expression in the ccRCC cells (data not shown). These results suggest that CEBPB promotes lipid accumulation in ccRCC primarily by inhibiting FAO, rather than by enhancing DNL, though DNL may work in some specific contexts.

Recently, CEBPB’s effects on CPT1A for FAO modulation have been reported in various diseases [26, 33, 65, 66], but mechanisms varied across tissues and experimental models. For example, CEBPB positively regulated CPT1A transcription in rat liver cells fed with a high-fat diet, nasopharyngeal carcinoma cells, and gastric cancer cells [33, 34, 66]. In another study, though, CEBPB negatively affected CPT1A expression indirectly in palmitic acid-stimulated BRL-3A rat liver cells [65]. Our results provide yet another mechanism of CEBPB’s regulation on CPT1A, through the direct transcriptional inhibition of GPD1L and intermediary metabolism. Therefore, our suggested mechanism may be specific to ccRCC, and its generalization to other tissues/conditions warrants further experimental validation. This specificity in ccRCC may have merits in exploiting the CEBPB pathway for the development of therapeutic agents.

While GPD1L is less studied than other GPD family proteins, its role in lipid metabolism and tumor progression is emerging with controversial functions [67, 68]. For instance, Yao et al. identified GPD1L as a tumor promoter in 786-O and Caki-1 cells [48]. Although the proposed GPD1L function in ccRCC is opposite to our conclusion, the lipid level changes (i.e., PE and PC decrease in GPD1/GPD1L double KD) and polar lipid involvement in ccRCC proliferation are consistent with our results. Along with our results, two independent studies support GPD1L’s tumor-suppressive role, especially via Akt inhibition and PINK1-mediated mitophagy enhancement [46, 47]. Although the negative correlation between GPD1L expression and Akt phosphorylation has been reported in esophageal squamous cell carcinoma and ccRCC [45, 47], the related mechanism remains unclear. One proposed mechanism is that GPD1L enhances AMPKα activity, which antagonizes the Akt phosphorylation [47, 69]. Our data suggest a new mechanism in which GPD1L regulates ether lipid synthesis and thereby modulates Akt phosphorylation within lipid rafts of the cell membrane. Additionally, this research also provided mechanistic insights linking GPD1L downregulation to reduced FAO and lipid accumulation through the GPD1L–ether lipids–Akt–CPT1A axis, further supported by patient cohorts showing GPD1L’s correlation with better prognosis. Some previous discrepancies might have been due to variations in the experimental conditions (e.g., KD or OE, use of cell culture media with or without pyruvate, use of Renca cells, GPD1L/GPD1 DKD). Overall, our results revealed a detailed and direct mechanism by which GPD1L and its metabolic products regulate lipid-related ccRCC tumorigenesis initiated by an epigenetically controlled TF.

Although it sounds quite natural to link dysregulated lipid metabolism to the *VHL* mutations, emerging evidence implicates VHL-independent or partially independent regulators of lipid metabolism in ccRCC, including JMJD6, GPX8, ring finger protein 20, and Kruppel-like factor 6 [13, 14, 70, 71]. Our CEBPB-GPD1L-ether lipid-Akt-CPT1A axis also demonstrated independence from *VHL* mutation, as neither OE nor KD of VHL affected CEBPB expression in Caki-1 and A498 cells. Given the epigenetic control of CEBPB via H3K27ac and H3K4me1, which are known to be environmentally responsive [72–75], CEBPB may link environmental factors to tumorigenesis. This might help explain delayed ccRCC development in patients with germline *VHL* mutations.

In conclusion, our research into lipid metabolism-related TFs in ccRCC revealed the epigenetically regulated and VHL-independent CEBPB-GPD1L-ether lipid-Akt-CPT1A axis. Significantly, the proposed mechanism presents how several specific elements in “Hallmarks of Cancer” [1] (e.g., epigenetics, metabolites, and signaling) can align in a single direct axis, as orchestrated by a transcription factor, CEBPB. The multiple components of this axis might be exploited as targets to develop new therapeutic agents for ccRCC.

## Supplementary information

Supplementary figures and Materials and Methods

Full and uncropped western blots

## Data Availability

The data for ccRCC-specific DACRs and TFs were extracted from the work of Terekhanova et al. [50]. The ChIP-seq data analyzed in this study have been deposited at Gene Expression Omnibus (GEO): GSE86095, GSE102095, and GSM935553. The scRNA-seq dataset GSE159115 analyzed in this study was obtained from 3CA (https://www.weizmann.ac.il/sites/3CA/). Data from TCGA can be retrieved from UCSC Xena (https://xenabrowser.net/). This paper analyzes existing, publicly available data, accessible at 10.1158/2159-8290.CD-17-0971.
