# Supplementary material for: Epigenetically-controlled CEBPB regulates kidney cancer tumorigenesis via GPD1L-mediated ether lipid synthesis
Source: Cell Death Dis. 2026 Jan 22;17(1):175. doi: 10.1038/s41419-025-08403-4 (PMC12877132; doi:10.1038/s41419-025-08403-4)
Supplement: Supplementary file 1 — Supplementary figures and Materials and Methods [file 41419_2025_8403_MOESM1_ESM.docx]

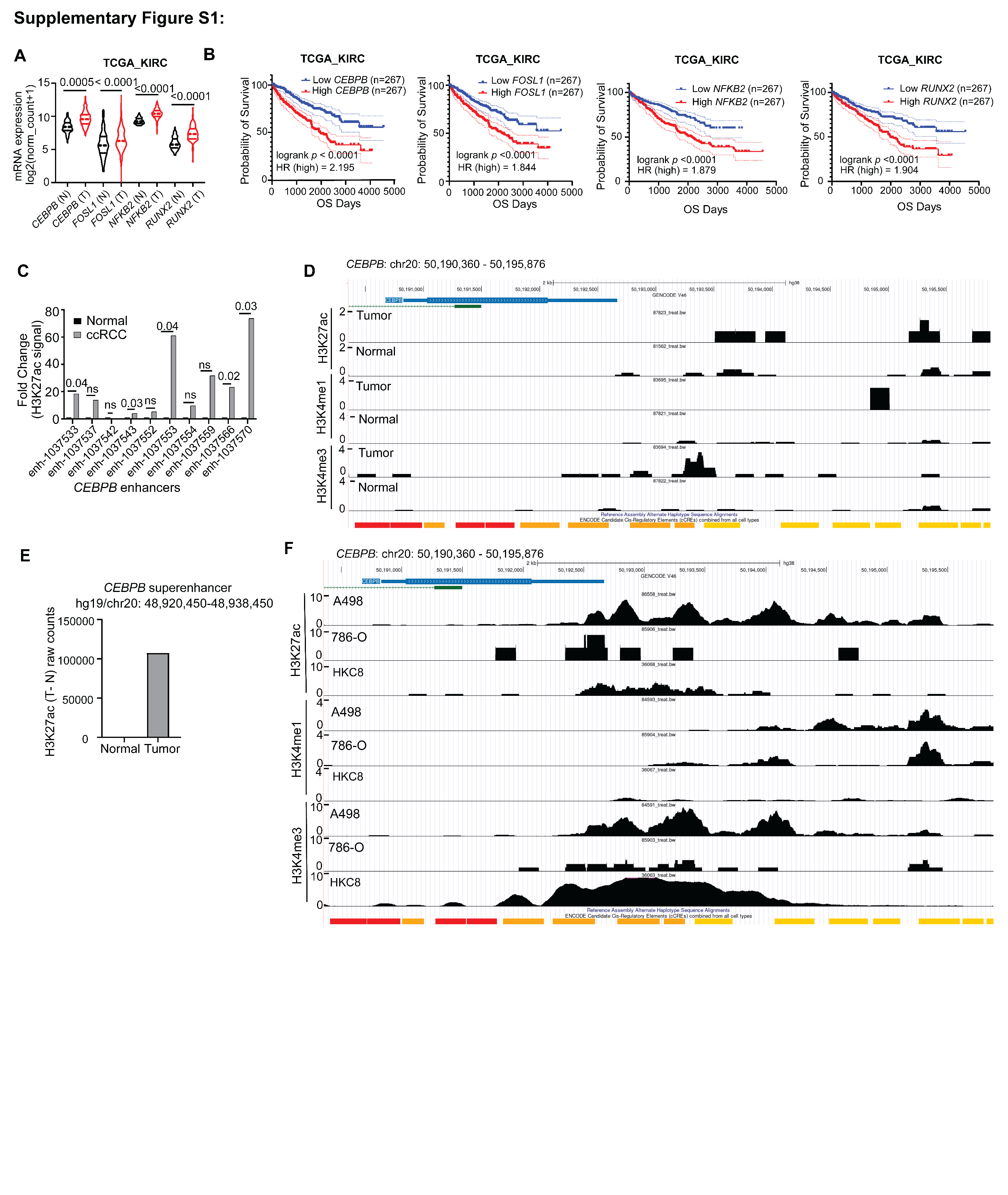


**Supplementary Figure S1:** **CEBPB is an epigenetically-regulated TF. A,** *FOSL1*, *CEBPB*, *NFKB*2, *RUNX2* expression in normal and ccRCC tissues in TCGA dataset. **B,** Kaplan-Meier analysis of survival data for ccRCC patients (TCGA-KIRC) based on mRNA expression of *FOSL1*, *CEBPB*, *NFKB*2, *RUNX2*. **C,** Fold difference of H3K27ac signal in altered promoters and enhancers regions of *CEBPB* in ccRCC tumor/normal pairs. **D,** H3K27ac, H3K4me1, and H3K4me3 ChIP-seq signals in ccRCC tumors and normal tissues shown for promoters and enhancers of *CEBPB* (GSM2293417, GSM2293413, GSM2293426, GSM2293414, GSM2293427, GSM2293415). **E,** A gained super-enhancer of *CEBPB* was identified in ccRCC tumor by the differential H3K27ac intensity between tumor and normal tissues. **F,** H3K27ac, H3K4me1, and H3K4me3 ChIP-seq show active *CEBPB* enhancers in ccRCC cells (786-O, A498) (GSM2723840, GSM2293336, GSM2293339, GSM2293334, GSM2293337, GSM2293332) compared with normal kidney cells (HKC8) (GSM1203230, GSM1203226, GSM1203228). *p*-value was calculated by Mann-Whitney U test for (**A**), log-rank (Mantel-Cox) test for (**B)**, paired *t*-test for **(C)**.

**
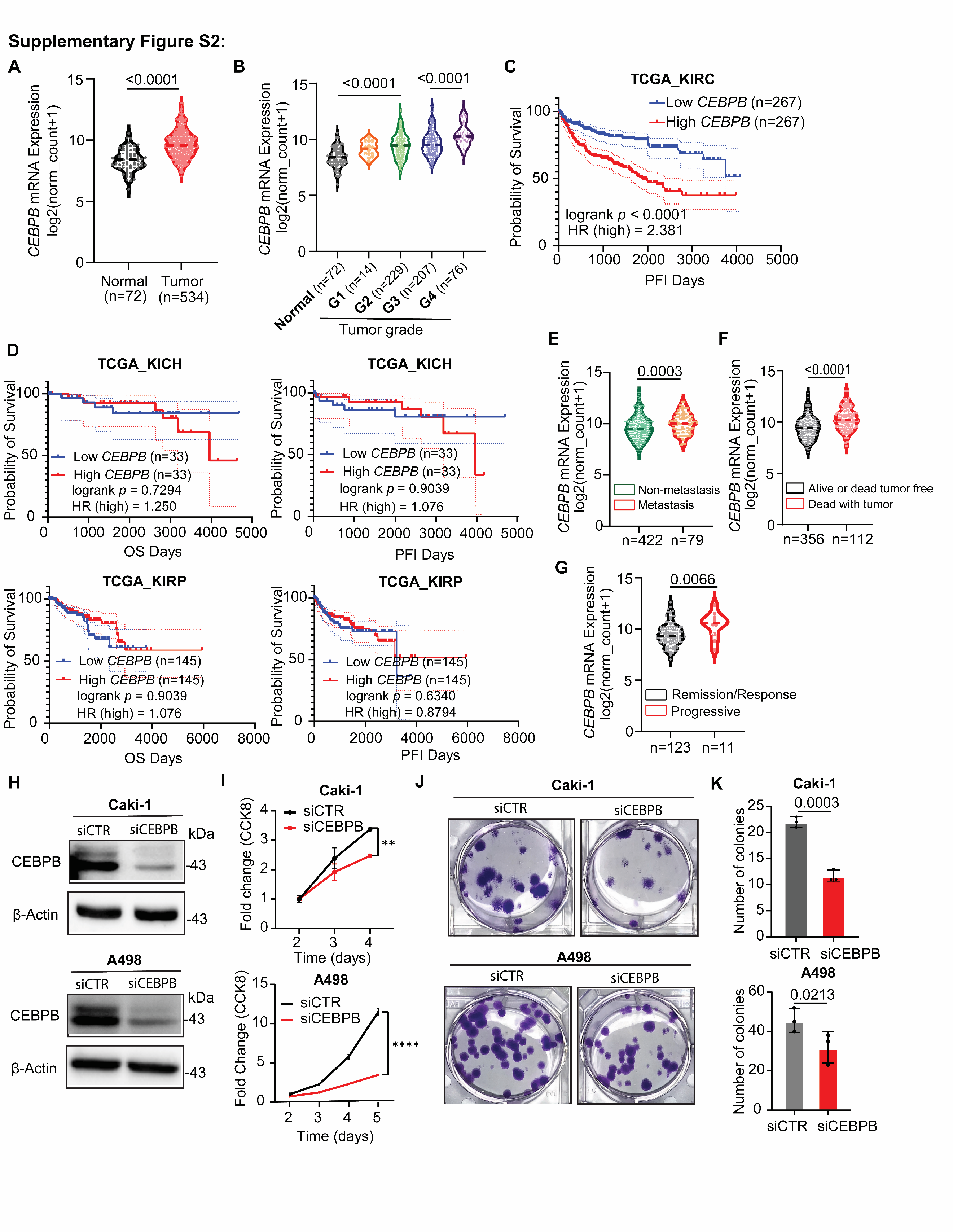
**

**Supplementary Figure S2:** **CEBPB is** **associated with higher grade and poor prognosis in ccRCC. A-B**, Violin plots for the mRNA expression levels of *CEBPB* according to normal vs. tumor (**A**) and neoplasm histologic grade (**B**) among ccRCC patients. **C**, Kaplan-Meier plots of PFI data for ccRCC patients based on *CEBPB* mRNA levels in TCGA-KIRC database (cutoff: median). **D**, Kaplan-Meier plots for the relationship between *CEBPB* mRNA expression and OS and PFI of patients in TCGA-KIRP, TCGA-KICH cohorts (cutoff: median). **E-G**, Violin plots for relationship between *CEBPB* mRNA expression and metastatic status (**E**), alive or dead related to tumor appearance (**F**), response or progression after primary therapies (**G**) in ccRCC patients. **H-K**, Immunoblotting analysis of CEBPB protein (**H**), relative growth rates measured by CCK8 kit (n = 3) (**I**), and colony formation and corresponding quantification data (n = 3) (**J and K**) in Caki-1 and A498 cell lines transduced with scramble siRNA or *CEBPB* siRNA. Error bars represent mean ± SD (n ≥ 3). *p*-value was calculated by Mann-Whitney U test for (**A**), (**B**), (**E**), (**F**), (**G**), log-rank (Mantel-Cox) test for (**C**) and (**D**), two-way ANOVA with Geisser-Greenhouse correction for (**I**), and un-paired *t*-test for panels (**K**). ^**^*p* < 0.002, ^****^*p* < 0.0001.

**
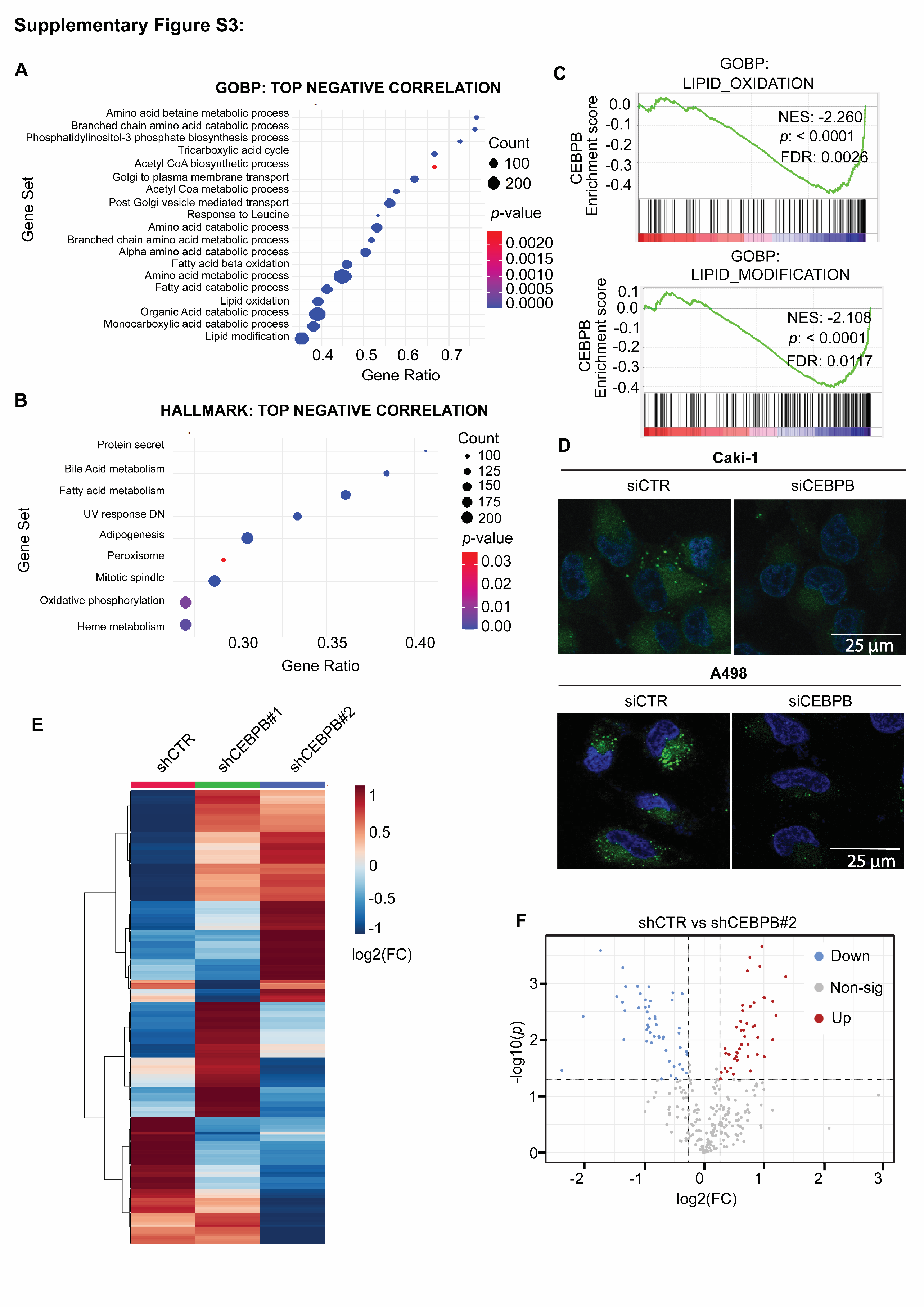
**

**Supplementary Figure S3: CEBPB KD decreased lipid content in ccRCC. A-B,** Top-ranked pathways show negative correlation with *CEBPB* mRNA expression in ccRCC by GSEA analysis with GO_BP (**A**) or hallmark (**B**) dataset. **C,** GSEA of the mRNA expression of *CEBPB* in TCGA-KIRC database and lipid metabolism. **D,** Representative pictures of 2 μM BODIPY 493/503 staining in serum-free medium for 15 min at 37℃, followed by staining with Hoechst from siCTR and siCEBPB Caki-1 and A498. **E,** Whole lipid profiles of shCTR and shCEBPB Caki-1 groups (n = 3). **F,** Volcano plot of altered lipid species from untargeted lipidomics for shCEBPB#2 vs. shCTR Caki-1. Criteria: |Fold change| ≥ 1.2 with *p*-value ≤ 0.05. *p*-value was calculated by *t*-test.

**
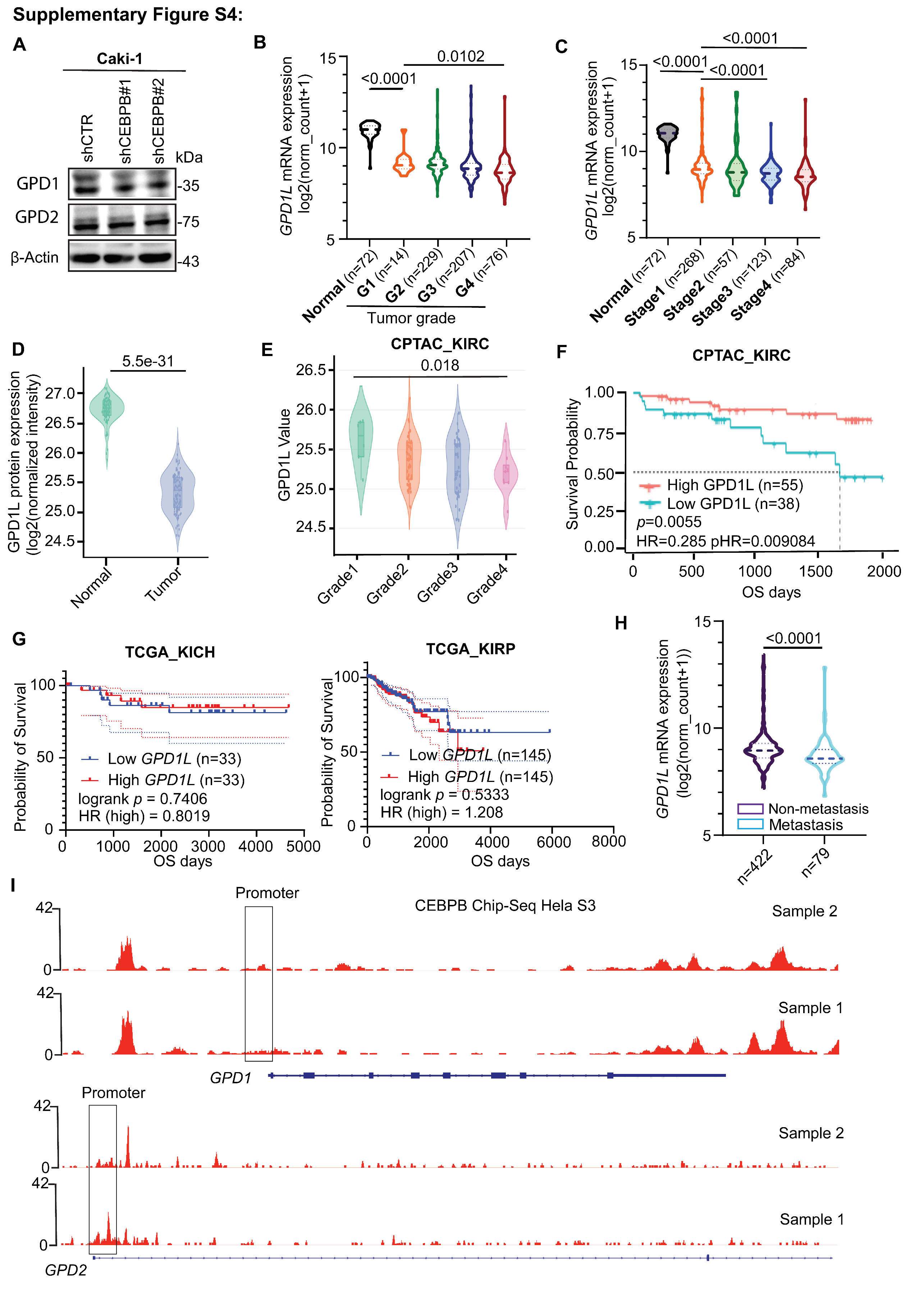
**

**Supplementary Figure S4:** **CEBPB negatively regulated GPD1L in ccRCC.**

**A,** Immunoblotting analysis of GPD1 and GPD2 expression responding to CEBPB KD in Caki-1 cells. **B-C,** Violin plots *GPD1L* mRNA expression according to neoplasm histologic grade (**B**) and histologic stage (**C**) among ccRCC patients. **D-E,** Violin plots GPD1L protein levels in normal and tumor tissues (**D**) and according to neoplasm histologic grade (**E**) among ccRCC patients (*https://pshlab.shinyapps.io/test22/*). **F,** Kaplan-Meier plots for the relationship between GPD1L protein levels and OS of ccRCC patients in CPTAC database (cutoff: optimal). **G,** Kaplan-Meier plots for the relationship between *GPD1L* mRNA expression and OS of patients in TCGA-KIRP, TCGA-KICH cohorts (cutoff: median). **H,** Violin plots for relationship between *GPD1L* mRNA expression and metastatic status in ccRCC patients. **I,** Analysis CEBPB ChIP-seq data in Hela-S3 cells with peaks in GPD1 and GPD2 regions by employing Integrative Genomic Viewer (IGV) (*https://igv.org/app/*). *p*-value was obtained by Mann-Whitney U test for (**B**), (**C**) and (**H**), log-rank (Mantel-Cox) test for (**G**).

**
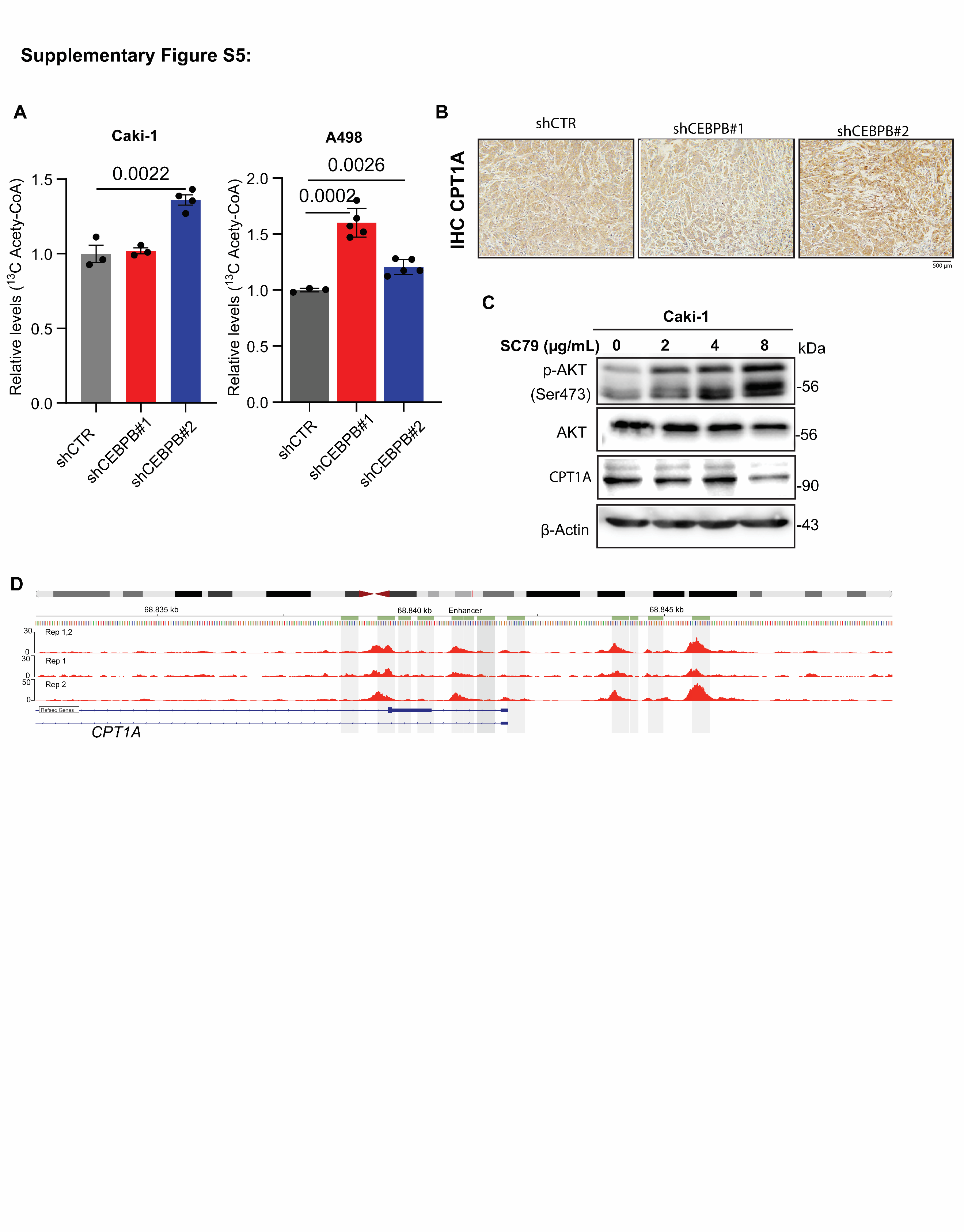
**

**Supplementary Figure S5:** **CEBPB/Akt signaling inhibited CPT1A expression in ccRCC.**

**A,** KD of CEBPB in Caki-1 and A498 cells increased ^13^C-labled acetyl-CoA (M+2 normalized to M+0) driven from ^13^C-labled palmitic acid. **B,** Presentative images of CPT1A IHC staining of tumors from *in vivo* xenografted mice with Caki-1 shCEBPB and shCTR. **C,** Expression of Akt, p-Akt (Ser473), and CPT1A in wild-type Caki-1 with DMSO or SC79 ranging from 2 µg/mL to 8 µg/mL treatment for 30 min, as detected by immunoblotting analysis. **D,** Analysis FOXO1 ChIP-seq data in HepG2 cells [(GEO:GSE170347)](https://www.ncbi.nlm.nih.gov/geo/query/acc.cgi?acc=GSE170347) with peaks in CPT1A regions by employing Integrative Genomic Viewer (IGV) (*https://igv.org/app/*). Error bars are means ± SD (n ≥ 3). *p*-value was obtained by un-paired *t*-test for (**A**).

**
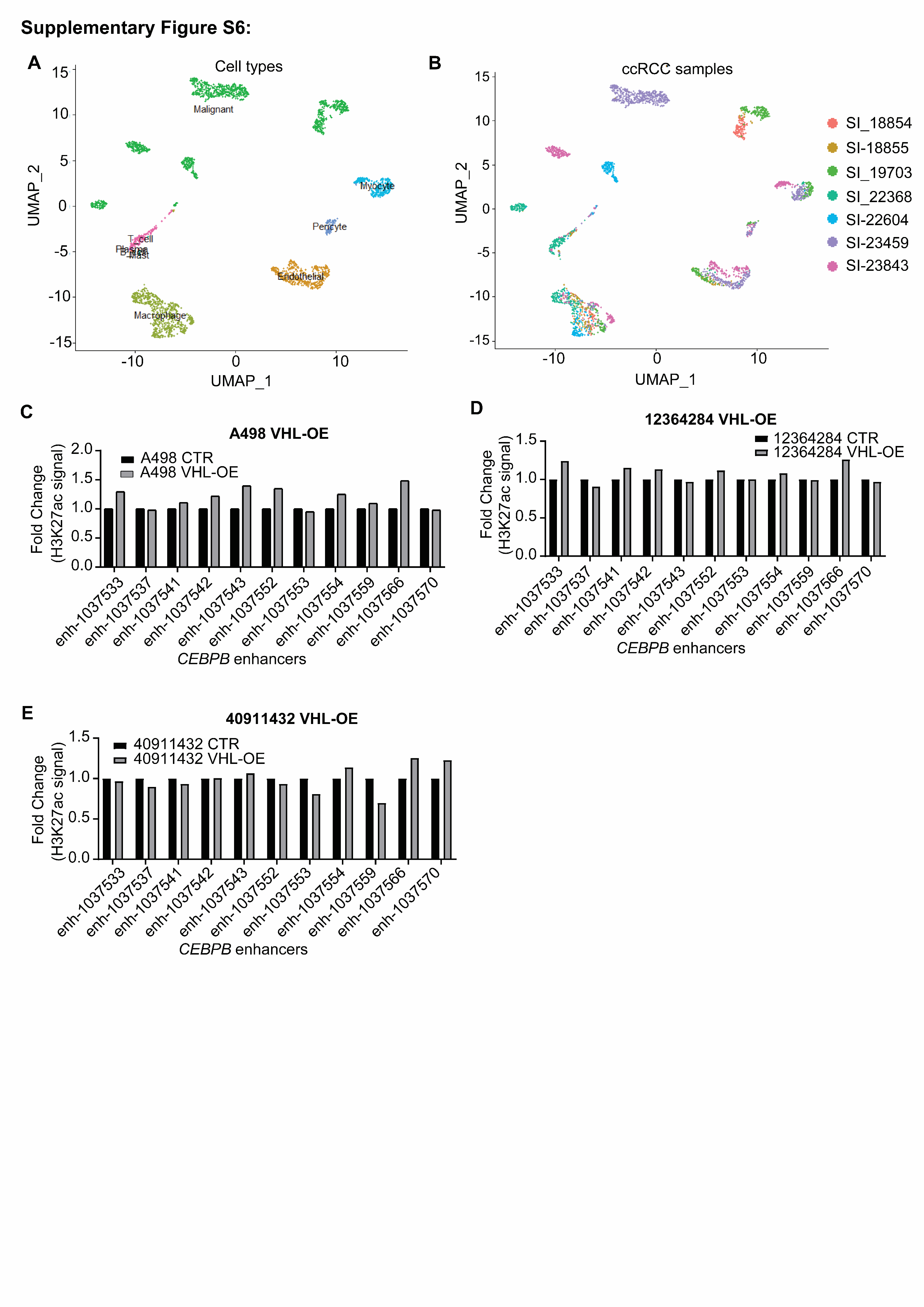
**

**Supplementary Figure S6: CEBPB expression is independent of *VHL* mutation status. A-B,** Cell clusters after integrative analysis of scRNA-seq data from 7 ccRCC samples. **C-E,** Enhancer H3K27 signals were unaltered by VHL restoration in A498 and ccRCC patient-derived cell lines (12364284 and 40911432).

**
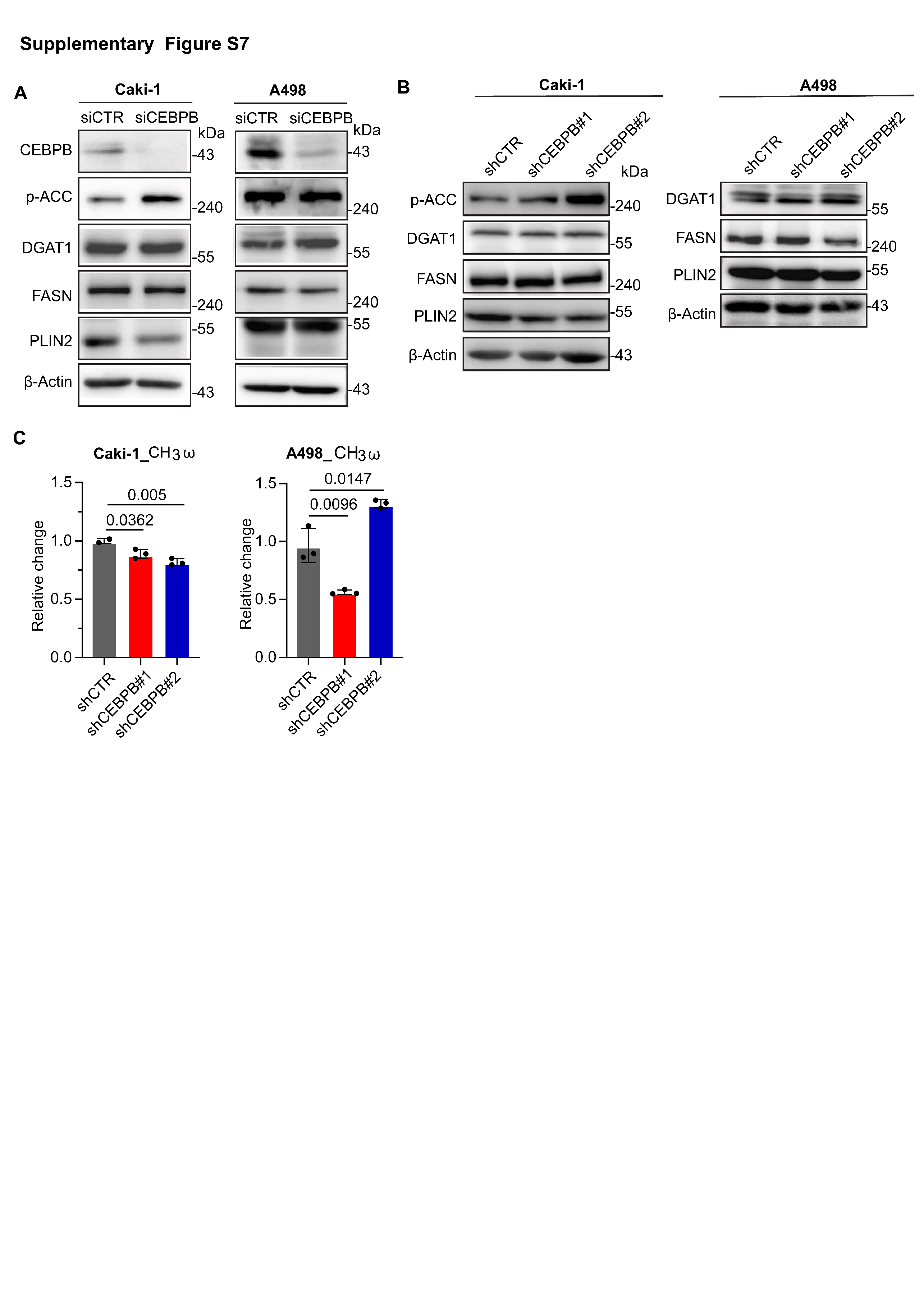
**

**Supplementary Figure S7: CEBPB could regulate DNL in ccRCC. A-B,** Immunoblotting analysis of CEBPB, CPT1A, DNL-related proteins (p-ACC and FASN), and TG/LD synthesis-related proteins (DGAT1 and PLIN2) in Caki-1 and A498 with siCEBPB (**A**) or shCEBPB (**B**). **C,** FA *de novo* synthesis (CH_3_ω) from U^13^C-glucose with NMR, and bar graphs for their relative levels normalized by protein mass comparing shCEBPB and shCTR Caki-1 and A498 (n = 3). Error bars represent mean ± SD (n = 3). *p*-value was calculated by un-paired *t*-test for (**C**).

ACC: acetyl-CoA carboxylase; FASN: fatty acid synthase; DGAT1: diacylglycerol O-acyltransferase 1; PLIN2: Perilipin-2

**
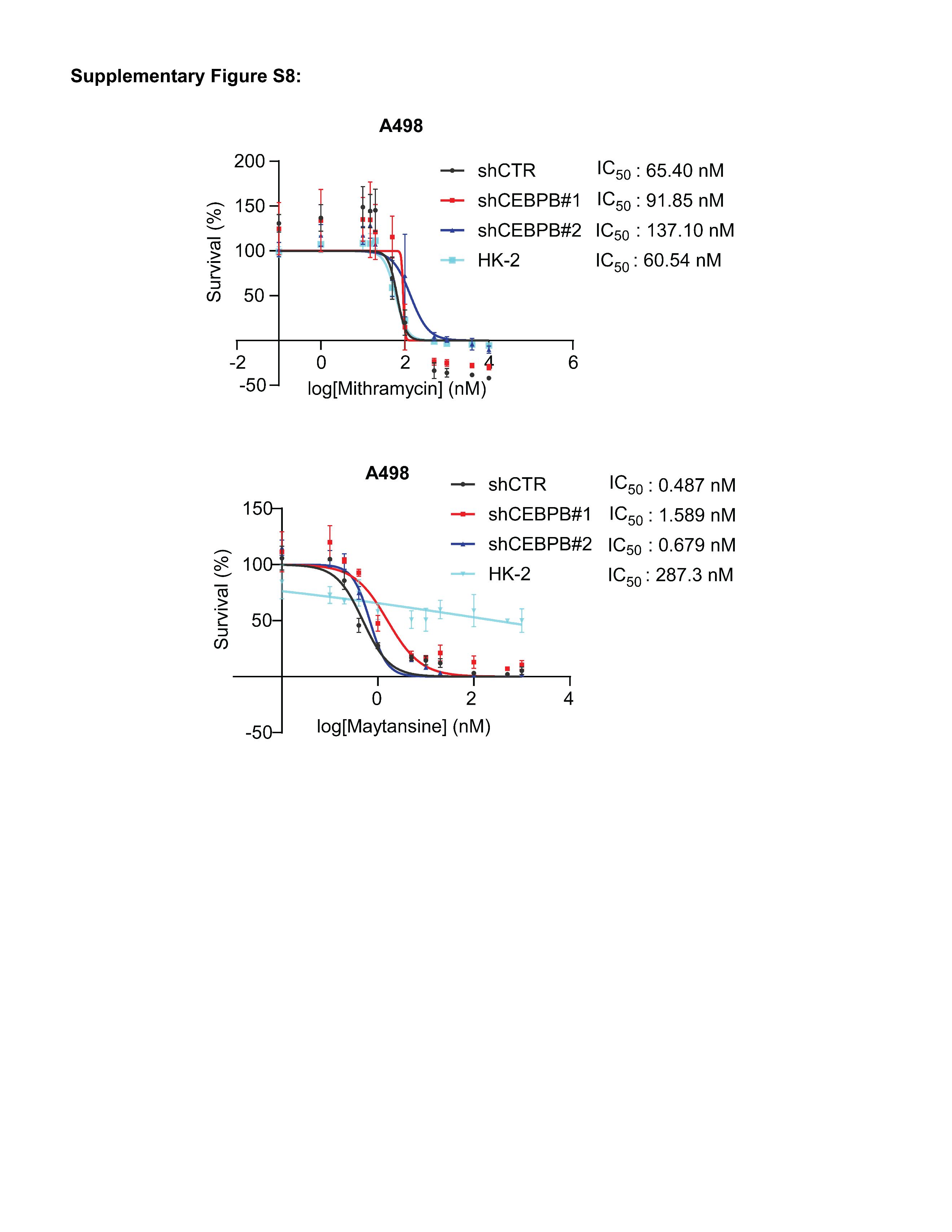
Supplementary Figure S8: Screening pharmacological inhibitor of CEBPB to suppresses ccRCC cell growth.** Effect of mithramycin and maytansine on the proliferation of kidney cancer cell lines A498 with shCTR, shCEBPB#1 or shCEBPB#2, and normal renal tubular epithelial cells HK-2. Cells were treated with range of concentrations of the drugs for 72 hours and cell viability was determined by SRB assay

**Supplementary Table S1:** Top 20 GO pathways have negative correlation to *CEBPB* and *FOSL1* in ccRCC

| **GENE** | **NAME** | **NES** | **FDR q-val** |
| --- | --- | --- | --- |
| *CEBPB* | GOBP_AMINO_ACID_BETAINE_METABOLIC_PROCESS | -2.60799 | 0 |
|  | GOBP_ORGANIC_ACID_CATABOLIC_PROCESS | -2.41559 | 5.44E-04 |
|  | GOBP_AMINO_ACID_CATABOLIC_PROCESS | -2.4091 | 3.63E-04 |
|  | GOBP_BRANCHED_CHAIN_AMINO_ACID_CATABOLIC_PROCESS | -2.36967 | 8.14E-04 |
|  | GOBP_BRANCHED_CHAIN_AMINO_ACID_METABOLIC_PROCESS | -2.33706 | 0.001972 |
|  | GOBP_PHOSPHATIDYLINOSITOL_3_PHOSPHATE_BIOSYNTHETIC_PROCESS | -2.30831 | 0.001829 |
|  | GOBP_FATTY_ACID_CATABOLIC_PROCESS | -2.28798 | 0.002659 |
|  | GOBP_FATTY_ACID_BETA_OXIDATION | -2.28072 | 0.002603 |
|  | GOBP_LIPID_OXIDATION | -2.26048 | 0.00256 |
|  | GOBP_GOLGI_TO_PLASMA_MEMBRANE_TRANSPORT | -2.24677 | 0.003299 |
|  | GOBP_POST_GOLGI_VESICLE_MEDIATED_TRANSPORT | -2.24074 | 0.003395 |
|  | GOBP_ALPHA_AMINO_ACID_CATABOLIC_PROCESS | -2.17997 | 0.007392 |
|  | GOBP_MONOCARBOXYLIC_ACID_CATABOLIC_PROCESS | -2.17197 | 0.007666 |
|  | GOBP_ACETYL_COA_METABOLIC_PROCESS | -2.12936 | 0.011347 |
|  | GOBP_ACETYL_COA_BIOSYNTHETIC_PROCESS | -2.11984 | 0.011751 |
|  | GOBP_TRICARBOXYLIC_ACID_CYCLE | -2.11155 | 0.011973 |
|  | GOBP_LIPID_MODIFICATION | -2.10763 | 0.011782 |
|  | GOBP_RESPONSE_TO_LEUCINE | -2.107 | 0.011127 |
|  | GOBP_AMINO_ACID_METABOLIC_PROCESS | -2.09803 | 0.011701 |
| *FOSL1* | GOBP_ORGANIC_ACID_CATABOLIC_PROCESS | -2.99638 | 0 |
|  | GOBP_FATTY_ACID_CATABOLIC_PROCESS | -2.92083 | 0 |
|  | GOBP_LIPID_OXIDATION | -2.79174 | 0 |
|  | GOBP_MONOCARBOXYLIC_ACID_CATABOLIC_PROCESS | -2.78722 | 0 |
|  | GOBP_FATTY_ACID_BETA_OXIDATION | -2.78356 | 0 |
|  | GOBP_AMINO_ACID_CATABOLIC_PROCESS | -2.75164 | 0 |
|  | GOBP_BRANCHED_CHAIN_AMINO_ACID_CATABOLIC_PROCESS | -2.71205 | 0 |
|  | GOBP_BRANCHED_CHAIN_AMINO_ACID_METABOLIC_PROCESS | -2.67684 | 0 |
|  | GOBP_ACYL_COA_METABOLIC_PROCESS | -2.61833 | 0 |
|  | GOBP_NUCLEOSIDE_BISPHOSPHATE_METABOLIC_PROCESS | -2.61301 | 0 |
|  | GOBP_AMINO_ACID_BETAINE_METABOLIC_PROCESS | -2.55942 | 0 |
|  | GOBP_TRICARBOXYLIC_ACID_CYCLE | -2.49253 | 1.34E-04 |
|  | GOBP_LIPID_MODIFICATION | -2.47878 | 1.24E-04 |
|  | GOBP_NUCLEOSIDE_BISPHOSPHATE_BIOSYNTHETIC_PROCESS | -2.41376 | 1.15E-04 |
|  | GOBP_L_AMINO_ACID_CATABOLIC_PROCESS | -2.40778 | 1.07E-04 |
|  | GOBP_PEROXISOME_ORGANIZATION | -2.40482 | 1.01E-04 |
|  | GOBP_TRICARBOXYLIC_ACID_METABOLIC_PROCESS | -2.40222 | 9.48E-05 |
|  | GOBP_SMALL_MOLECULE_CATABOLIC_PROCESS | -2.3995 | 8.95E-05 |
|  | GOBP_DICARBOXYLIC_ACID_METABOLIC_PROCESS | -2.36693 | 2.13E-04 |

**Supplementary Table S2: Key resources tables**

| **REAGENT or RESOURCE** | **SOURCE** | **IDENTIFIER** |  |
| --- | --- | --- | --- |
| **Antibodies** | | | |
| β-actin | Santa Cruz Biotechnology | Cat# sc-47778 |  |
| CEBPB | Abcam | Cat# ab32358 |  |
| GPD1L | Proteintech Group | Cat# 17263-I-AP |  |
| GPD1 | Santa Cruz Biotechnology | Cat# sc-390379 |  |
| GPD2 | Santa Cruz Biotechnology | Cat# sc-393620 |  |
| Akt1 | Abcam | Cat# ab238477 |  |
| Phospho-Akt (Ser473) | Abcam | Cat# ab81283 |  |
| CPT1A | Cell Signaling Technology | Cat# 12252 |  |
| CPT1A | ABclonal | Cat# A5307 |  |
| VHL | Cell Signaling Technology | Cat# 68547 |  |
| HIF-1α | Bethyl Laboratories | Cat# A300-286A |  |
| HIF-2α (D9E3) | Cell Signaling Technology | Cat# 7096 |  |
| Goat anti-mouse IgG (H+L) Secondary Antibody, HRP | Invitrogen | Cat# 31430 |  |
| Goat anti-rabbit IgG (H+L) Secondary Antibody, HRP | Invitrogen | Cat# 31460 |  |
| **Chemicals, peptides, and recombinant proteins** | | | |
| U^13^C-glucose | Cambridge Isotope Laboratories, Inc. | Cat# CLM-1396-PK |  |
| U^13^C-palmitate | Cambridge Isotope Laboratories, Inc. | Cat# CLM-3943-  0.5 |  |
| BODIPY 493/503 | ThermoFisher Scientific | Cat# D3922 |  |
| Oil Red | Sigma-Aldrich | Cat# O0625 |  |
| Matrigel | BD Biosciences | Cat# 356234 |  |
| DMEM | WELGENE Inc. | Cat# LM001-05 |  |
| KSFM | Thermo Fisher Scientific Inc | Cat# 17005042 |  |
| Fetal bovine serum FBS | WELGENE Inc. | Cat# S001-07 |  |
| Penicillin-streptomycin | WELGENE Inc. | Cat# LS202-02 |  |
| **Critical commercial assays** | | | |
| CCK8 (Cell Counting Kit-8) | Dojindo | Cat# CK04-11 |  |
| Chemiluminescent reagent ECL Solution | Abfrontier WESTSAVE | Cat# F-QC0106 |  |
| SYBR green-based detection (iTaq TM Universal  SYBR Green Supermix) | Bio-Rad, USA | Cat# 172-5120 |  |
| TOPreal SYBR Green qPCR High-Rox PreMIX | Enzynomics | Cat# RT501S, |  |
| High-Capacity cDNA Reverse Transcription Kit | Applied Biosystems | Cat# 4368814 |  |
| Myco-Read^TM^ Mycoplasma Detection Kit | Biomax Ltd. | Cat# SMD0173 |  |
| **Experimental models: Cell lines** | | | |
| HEK293T | ATCC | Cat# ATCC-CRL-  3216 |  |
| Caki-1 | Korean Cell Line Bank | Cat# 30046 |  |
| A498 | Korean Cell Line Bank | Cat# 30044 |  |
| HK-2 | Korean Cell Line Bank | Cat# 22190 |  |
| **Experimental models: Organisms/strains** | | | |
| Tissue array of kidney carcinoma and matched adjacent  tissue | US Biomax, Inc., | KD485 |  |
| Male BALB/c nude mice | Orient Bio Laboratory Animal Research Center Co., Ltd | N/A |  |
| **Oligonucleotides** | | | |
| siRNA targeting sequence: targeting CEBPB  GACAAGCACAGCGACGAGU | Bioneer | Cat# 1051-1 |  |
| shRNA targeting sequence: targeting CEBPB  sh#1: GCAATCGGTTTAAACATGGCT  sh#2: AGCACAGCGACGAGTACAAGA | GeneCopoeia | Cat#  HSH067167  -LVU6GP |  |
| shRNA Control  GCTTCGCGCCGTAGTCTTA | GeneCopoeia | Cat#  CSHCTR001  -LVU6GP |  |
| siRNA CPT1A | Bioneer | Cat# 1374-1 |  |
| siRNA VHL | Bioneer | Cat# 7428-1 |  |
| *GAPDH* primers:  Forward: 5′-GAGTCAACGGATTTGGTCGT-3′,  Reverse: 5′-TTGATTTTGGAGGGATCTCG-3′ | This paper | N/A |  |
| *CEBPB* primers:  Forward: 5′-GGCCCTGAGTAATCGCTTAAAG-3′,  Reverse: 5′-TCCCAAAATATACAGACGCCTC-3′ | This paper | N/A |  |
| *CPT1A* primers:  Forward: 5′-GAAGATGGCAGAAGCTCACC-3′,  Reverse: 5′-TGGCGTACATCGTTGTCAT-3 | This paper | N/A |  |
| *GPD1L* primers:  Forward: 5′-CGCTGGGAATCACCCTCATC-3′,  Reverse: 5′-AATGTTGGCTCCCATCAGCA-3′ | This paper | N/A |  |
| Predicted *GPD1L* promoter region 1:  Forward: 5′-GGACTATAGGTGCGTGCCA-3′  Reverse: 5′-GATCACTTGAGCCCAGGAGT-3′ | This paper | N/A |  |
| Predicted *GPD1L* promoter region 2:  Forward: 5′-ACTCCTGGGCTCAAGTGATC-3′  Reverse: 5′-CAGTACCTGCATTTCAAGGATGA-3′ | This paper | N/A |  |
| Predicted *GPD1L* promoter region 3:  Forward: 5′-AAGTCTGGTCGGCTTGGAGAG-3′  Reverse: 5′-TGGAGCAGACCCAACCAG-3′ | This paper | N/A |  |
| Predicted *GPD1L* promoter region 4:  Forward: 5′-ATGAGGATGGAGTGCAGCC-3′  Reverse: 5′-GCCACTCCCAAGAACCACT-3′ | This paper | N/A |  |
| **Recombinant DNA** | | | |
| PLP-1 | N/A | N/A |  |
| PLP-2 | N/A | N/A |  |
| VSV-G | N/A | N/A |  |
| pCMV6-Entry-Myc-DDK-tag carried GPD1L | Origene | Cat# RC206131 |  |
| pCMV6-Entry-Myc-DDK-tag carried VHL | Origene | Cat#: RC216151 |  |
| pCMV6-Entry Mammalian Expression Vector (control) | Origene | Cat#: PS100001 |  |
| GLuc-ON™ Promoter Reporter Clones pEZX-PG04  carried GPD1L promoter | GeneCopoeia | Cat# HPRM71844-PG04 |  |
| **Software and algorithms** | | | |
| Topspin | Bruker | Ver. 3.6.3 |  |
| MZmine | N/A | Ver 3.7.2 |  |
| R | N/A | Ver. 4.2.3 |  |
| GraphPad Prism | N/A | Ver. 10.2.1 |  |
| ImageJ | N/A | N/A |  |
| FlowJo | N/A | Ver. 7.6.2 |  |

**Materials and methods**

**Cell culture**

HEK293T (Cat# ATCC-CRL-3216, ATCC, Manassas, VA, USA) and the human ccRCC cell lines Caki-1 (Cat# 30046, Korean Cell Line Bank, Seoul, Korea) and A498 (Cat# 30044, Korean Cell Line Bank) were cultured in high-glucose DMEM (Cat# LM001-05, WELGENE Inc., Gyeongsan, Korea) supplemented with 10% fetal bovine serum FBS (Cat# S001-07, WELGENE Inc.) and 1% penicillin-streptomycin (Cat# LS202-02, WELGENE Inc.). Human normal renal tubular epithelial cell line HK-2 (Cat# 22190, Korean Cell Line Bank) was cultured in KSFM (Cat# 17005042, Thermo Fisher Scientific Inc, Waltham, MA, USA). Mycoplasma contamination was routinely tested using Myco-Read^TM^ Mycoplasma Detection Kit (Cat# SMD0173, Biomax Ltd., Guri, Korea).

**Plasmids, lentiviral production, and transduction**

For genetic KD, siRNA oligonucleotide targeting CEBPB (Cat# 1051-1, GACAAGCACAGCGACGAGU) was ordered from BIONEER Co. (Daejeon, Korea). Lentiviral vectors expressing shCEBPB (Cat# HSH067167-LVU6GP; #1 GCAATCGGTTTAAACATGGCT; #2 AGCACAGCGACGAGTACAAGA), including a shCTR vector (Cat# CSHCTR001-LVU6GP; GCTTCGCGCCGTAGTCTTA) were obtained from GeneCopoeia, Inc. (Rockville, MD, USA). Lentivirus was produced by transfecting HEK293T cells with lentiviral shRNA vectors, PLP-1, PLP-2, and VSV-G using polyethylenimine (Cat# 408727, Sigma-Aldrich, St. Louis, MO, USA). Lentivirus particles were harvested at 48 h post-transfection and concentrated using Lenti-X-Concentrator (Cat# 631331, Takara Bio Inc., Shiga, Japan). For viral transduction, the cells were incubated with lentivirus in the presence of 10 µg/mL polybrene (Cat# TR-1003, Sigma-Aldrich) for 24 h, followed by antibiotic selection (puromycin, Cat# P8833, Sigma-Aldrich) for 4-5 days. Live cells were seeded for single-cell selection in a 96-well plate. The single-cell colony was expanded, KD efficiency was evaluated by RT-qPCR and immunoblotting analysis, and the cells were cultured for further experiments.

**Xenograft mouse**

BALB/c nude mice (male, 6 weeks old) were purchased from Orient Bio Laboratory Animal Research Center Co., Ltd. (Seoul, Korea). Mice were kept (*n* = 5 per cage) for a week to acclimatize before carrying out the experiments. Mice are randomly divided into the three groups: control groups with shCTR Caki-1 inoculation (n = 8), CEBPB KD with shCEBPB#1 Caki-1 inoculation (n = 10), shCEBPB#2 Caki-1 inoculation (n = 8) (1). Caki-1 (5 × 10^6^) cells with stable expression of shCTR, shCEBPB#1, and shCEBPB#2 were mixed in a 1:1 ratio with Matrigel Basement Membrane Matrix High Concentration (Cat# 354248, Corning, Corning, NY, USA) in 200 µL DPBS (Cat# LB 001-02, WELGENE Inc.) and subcutaneously implanted into the flank of mice. Seven days after implantation, the tumor size was monitored using a Vernier caliper to measure the length (L), width (W), and height (H) of each tumor every 5-7 days. Tumor volume was identified using the formula V = (L x W x H)/2. Mice that showed no tumor growth due to a technical error were excluded. The whole procedure was deliberated and accepted by Seoul National University IACUC (Number: SNU-240417-1).

**RT-qPCR**

Total RNA was extracted using an Easy-spin RNA extraction kit (Cat# 17221, iNtRON Biotechnology, Inc., Seongnam, Korea). One microgram of RNA was used to synthesize cDNA using a High-Capacity cDNA Reverse Transcription Kit (Cat# 4368814, Applied Biosystems, Inc., Waltham, MA, USA). qPCR was performed in triplicate in an Applied Biosystems 7300 PCR machine with TOPreal SYBR Green qPCR High-Rox PreMIX (Cat# RT501S, Enzynomics Co., Ltd., Daejeon, Korea) or Thermo QuanStudio TM 5 with iTaq Universal SYRB^®^ Green Supermix (Cat# 1725121, Bio-Rad Laboratories, Inc., Hercules, CA, USA). The ΔCt values were calculated by normalization to *GAPDH*. *GAPDH*, *CEBPB*, *CPT1A*, and *GPD1L* primers were obtained from BIONEER Co. (Daejeon, Korea). *GAPDH*: Forward: 5′-GAGTCAACGGATTTGGTCGT-3′, Reverse: 5′-TTGATTTTGGAGGGATCTCG-3′; *CEBPB*: Forward: 5′-GGCCCTGAGTAATCGCTTAAAG-3′, Reverse: 5′-TCCCAAAATATACAGACGCCTC-3′; *CPT1A*: Forward: 5′-GAAGATGGCAGAAGCTCACC-3′, Reverse: 5′-TGGCGTACATCGTTGTCAT-3′; *GPD1L*: Forward: 5′-CGCTGGGAATCACCCTCATC-3′, Reverse: 5′-AATGTTGGCTCCCATCAGCA-3′

**Immunoblotting analysis**

The cells were scraped and lysed in RIPA buffer containing Xpert Phosphatase Inhibitor Cocktail Solution (100×) (Cat# P3200-001, GenDEPOT, Altair, TX, USA) and Protease Inhibitor Cocktail (100×) (Cat# ab271306, Abcam, Cambridge, UK). After 30 min of incubation on ice, the lysates were centrifuged at 13,000 × g, 4℃ for 20 min. The supernatant was collected and protein content was quantified and normalized using Pierce^TM^ BCA Protein Assay Kit (Cat# 23225, Thermo Fisher Scientific). The supernatants were mixed with SDS buffer 5× and incubated at 100℃ for 5 min in a heating block. The prepared samples were loaded onto 6% - 13% SDS-PAGE gels and transferred to a nitrocellulose membrane. The membrane was blocked with 5% BSA in TBS-T for 1 h at room temperature (RT), followed by incubation with primary antibodies (diluted 1:1000) for overnight at 4℃. These antibodies were used in experiments: β-actin (Cat# sc-47778, Santa Cruz Biotechnology, Inc., Dallas, TX, USA), CEBPB (Cat# ab32358, Abcam), GPD1L (Cat# 17263-I-AP, Proteintech Group, Inc., Rosemont, IL, USA), GPD1 (Cat# sc-390379, Santa Cruz Biotechnology), GPD2 (Cat# sc-393620, Santa Cruz Biotechnology), Akt1 (Cat# ab238477, Abcam), Phospho-Akt (Thr308) (Cat# 4056, Cell Signaling Technology, Inc., Danvers, MA, USA), Phospho-Akt (Ser473) (Cat# ab81283, Abcam), CPT1A (Cat# 12252, Cell Signaling Technology), VHL (Cat# 68547, Cell Signaling Technology), HIF-1α (Cat# A300-286A, Bethyl Laboratories, Inc., Montgomery, TX, USA), HIF-2α (Cat# 7096, Cell Signaling Technology). For detection, goat anti-mouse IgG (H+L) Secondary Antibody, HRP (Cat# 31430, Invitrogen, Thermo Fisher Scientific), goat anti-rabbit IgG (H+L) Secondary Antibody, HRP (Cat# 31460, Invitrogen, Thermo Fisher Scientific) were used, followed by exposure to a chemiluminescent reagent (West Save Star, Cat# LF-QC0106, Abfrontier, Seoul, Korea). Imaging of the blots was performed using an Image Analyzer (SOLO.6x)-Chemi DOC (VILBER LOURMAT, Collégien, France). Full, uncropped blots are provided in Supplementary Material.

**Cell proliferation and colony assay**

Cell proliferation was performed using CCK8 reagent (Cat# CCK-3000, Dojindo, Kumamoto, Japan). Cells were plated at 1.5 x 10^3^ cells per well in 96-well plates, and after 24, 48, 72, and 96 h, the CCK8 assay was performed following the manufacturer’s instructions.

Cell viability and cytotoxicity screening were determined by sulforhodamine B (SRB) assay (2). To treat cells with Akt Activator II, SC79 (Cat# 123871, Sigma-Aldrich) and plasmalogen PC (18:0p/18:1) (Cat# 852467C, Avanti Polar Lipids, LLC., Alabaster, AL, USA), cell viability was determined by SRB assay after 72 h of treatment. For cytotoxicity screening, A498 (shCTR, shCEBPB#1, shCEBPB#2) (6 x 10^3^) and HK-2 (7 x 10^3^) cells per well were seeded in 96-well plate. Mithramycin (Cat# M6891, Sigma-Aldrich), BIO8898 (Cat# HY-122663A, MedChem Express, Monmouth Junction, NJ, USA), maytansine (Cat# SML3451, Sigma-Aldrich) were dissolved in DMSO at stock concentration of 10 mM. These drugs were treated to cells with the range of concentration and final DMSO in medium was 0.1 %. After 72 h incubation in 37℃, 5 % CO_2_, cell viability was determined by SRB assay. Cells were fixed with 10% trichloroacetic acid (TCA, Cat# T6399, Sigma-Aldrich) for 30 min at 4℃ followed by RT drying. Fixed cells were stained with SRB solution in 1% acetic acid (Cat# A1756, Samchun Chemical Co., Ltd., Seoul, Korea) for 2 h at RT. Then cells were washed under tap water followed by 1% acetic acid wash several times and were dried before being extracted with Tris-HCl 1.5 M (pH 8.8) (200 µL/well/96-well plate) for 2 h shaking at RT. The SRB staining intensity was measured using a VersaMax microplate reader (Molecular Devices, LLC., San Jose, CA, USA) at 515 nm. IC_50_ was identified using nonlinear regression (log(inhibitor) vs normalized response - Variable slope) of the GraphPad Prism 10.2.1 software.

For colony formation, 100 cells per well were plated in 6-well plates and cultured in growth medium for 2 weeks, and the medium was changed every 3 days. The cells were then stained with 0.5% crystal violet (Cat# C2460, TCI, Tokyo, Japan) in 20% methanol (Cat# 4257, DUKSAN PURE CHEMICALS Co., Ltd., Ansan, Korea) for 5 min. The number of colonies was quantified by using the ImageJ software.

**Lipid droplet staining**

For ORO staining, cells were seeded in 6-well plates for 48 h to reach 90% confluency. The cells were washed with PBS and fixed with 10% formalin (Cat# HT501128, Sigma-Aldrich) for 15 min before staining with 0.5% ORO (Cat# O0625, Sigma-Aldrich) in propylene glycol (PEG, Cat# 609, DUKSAN PURE CHEMICALS) for 20 min. The stained cells were washed with tap water before counterstaining with hematoxylin (Cat# ab220365, Abcam) for 2 min. The cells were mounted with an aqueous mounting medium (Cat# ab64230, Abcam) and examined under light microscopy (BX50, Olympus Co., Tokyo, Japan).

For BODIPY 493/503 (Cat# D3922, Life technology, Thermo Fisher Scientific) staining, cells were washed with PBS and incubated in 2 μM BODIPY 493/503 in serum-free medium for 15 min at 37℃ before fixing with 4% paraformaldehyde (Cat# PC2031-100-00, BIOSESANG, Yongin, Korea), followed by staining with Hoechst (Cat# 33342, Thermo Fisher Scientific) for nuclei. The fixed cells were then visualized using confocal scope TCS8 (Leica, Wetzlar, Germany).

For Nile red staining, cells were washed with PBS and incubated in 5 μM Nile red in PBS for 20 min, and stained cells were trypsinized to measure intensity using flow cytometer (FACSCalibur, BD Biosciences, San Jose, CA, USA) with the FL2 channel. The fluorescence intensity was quantified and visualized by using FlowJo version 7.6.2.

**Immunohistochemistry (IHC) experiment**

A kidney carcinoma tissue microarray slide containing 48 samples (37 cases of clear cell carcinoma, two cases of papillary renal cell carcinoma, one case of sarcomatoid carcinoma, and eight cases of normal tissues) and a single core per case (Cat# KD485, US Biomax, Rockville, MD, USA) embedded in paraffin was utilized to check CEBPB expression. Samples from shCEBPB Caki-1 xenografted mouse were embedded in paraffin and then spliced to slide with 3 µm thickness for IHC.

The slides were immunolabeled with CEBPB antibody (Cat# ab32358, Abcam), CPT1A (Cat# A5307, ABclonal, Woburn, MA, USA), and GPD1L (Cat# 17263-I-AP, Proteintech) using heat-induced antigen retrieval and standard IHC procedure. After incubation with primary antibody (1:200 in 0.5% BSA) overnight at 4℃, the slides were incubated with secondary HRP and 3,3’-DAB substrate as the chromogen (Cat# K5007, Dako Real Envision Detection System, Peroxidase/DAB^+^, Agilent Technologies, Santa Clara, CA, USA). Finally, slides were lightly counter-stained with hematoxylin (Cat# ab220365, Abcam) for 3 min, and mounted with organic mounting solution (Permount™ Mounting Medium (Cat# SP15-500, Fisher Chemical): xylene (Neo-clear, Cat# 1.09843, Sigma-Aldrich) (1:1)), and dried at RT. Slides were observed using PerkinElmer Vectra 3.0, and images were visualized using Phenochart 1.0.12.

**Chromatin immunoprecipitation (ChIP)-PCR**

After reaching 90% confluence, Caki-1 cells were cross-linked with 1% formaldehyde for 10 min and quenched using 100 mM glycine for 5 min at RT. The cell pellet was resuspended in lysis buffer and sonicated (10 s pulse and 10 s rest, 30 cycles at 60% amplification). A/G-agarose beads (Cat# sc-2003, Santa Cruz Biotechnology, Inc.) were used for immunoclearing (2 h at 4℃) and immunoprecipitation. Immunoprecipitation was performed using the antibody against CEBPB (Cat# ab32358, Abcam) and anti-rabbit IgG (Cat# 02-6102, Invitrogen) as a negative control. Chromatin was recovered by incubation with RNase A (Cat# IBS-BR003, iNtRON Biotechnology, Inc.). RNA and proteins were removed by using Proteinase K (Cat# E0491, Thermo Fisher), and then DNA purification was performed manually. The interaction between CEBPB and predicted *GPD1L* promoter was detected using PCR with primer sequences. Region 1: Forward: 5′-GGACTATAGGTGCGTGCCA-3′, Reverse: 5′-GATCACTTGAGCCCAGGAGT-3′; Region 2: Forward: 5′-ACTCCTGGGCTCAAGTGATC-3′, Reverse: 5′-CAGTACCTGCATTTCAAGGATGA-3′; Region 3: Forward: 5′-AAGTCTGGTCGGCTTGAGAG-3′, Reverse: 5′-TGGAGCAGACCCAACCAG-3′; Region 4: Forward: 5′-ATGAGGATGGAGTGCAGCC-3′, Reverse: 5′-GCCACTCCCAAGAACCACT-3′.

**Luciferase reporter assay**

The GPD1L promoter reporter constructed into the GLuc-ON™ Promoter Reporter Clones pEZX-PG04 (Cat# HPRM71844-PG04) was obtained from GeneCopoeia, Inc.. The pEXZ-PG04-*GPD1L* promoter reporter plasmid was transfected into the cells using Lipofectamine 3000 (Cat# L3000015, Thermo Fisher Scientific). After 48 h post-transfection, culture medium was collected and tested using the Secrete-Pair^TM^ Dual Luminescence Assay Kit (Cat# LF031, GeneCopoeia, Inc.) following the manufacturer’s instructions and a Luminometer (Centro LB960, Berthold Technologies GmbH & Co. KG, Bad Wildbad, Germany).

**Promoter binding-site prediction**

Promoter of GPD1L (-1000 bp to +100 bp) was identified using [*https://epd.expasy.org/*](https://epd.expasy.org/)*.* Prediction of putative binding-site of CEBPB in GPD1L promoter was performed using *https://alggen.lsi.upc.es/* with five maximum matrix dissimilarity rate.

**NMR experiments**

Cells were plated at 70-80% confluency in 100 mm dished overnight. The following day, the medium was changed to 25 mM U^13^C-glucose (Cat# CLM-1396-5, Cambridge Isotope Laboratories, Inc., Andover, MA, USA) containing 10% dialyzed FBS (Cat# 26-400-044, Gibco, Thermo Fisher Scientific) and cultured for 6 h before extraction. After culturing the cells in specific experiment conditions, the cells were washed three times with chilled PBS, scraped, transferred into the new 2.0 EP tubes, and then extracted using the freeze-thaw cycle (three times) in DW: MeOH: CHCl_3_ (1:2:2). The mixture was centrifuged at 15,000 × g at 4℃ for 20 min. The upper and lower phases were separated, dried using a vacuum evaporator, and stored at -80℃ until further analysis. Hydrophobic extracts were dissolved in CDCl_3_ (Cat# 151823, Sigma-Aldrich). The 2D HSQC spectra were obtained using 800 MHz NMR Bruker Avance (Bruker Biospin GmbH, Rheinstetten, Germany). Peak intensity of CH_3_ω and TG were quantified and normalized to the protein mass.

**LC/MS/MS untargeted lipidomics**

The cell extraction procedure for LC/MS/MS followed the NMR protocol. Dried hydrophobic extracts were dissolved in 10 µL of a mixture of CHCl_3_: MeOH (2:1), and then diluted in 40 µL of ACN: IPA: DW (65:30:5), and centrifuged at 15,000 × g at 4℃ for 5 min. BEH C18 column was used with buffer A (10 mM Ammonium Formate in ACN: DW = 6:4 with 0.1% formic acid) and buffer B (10 mM Ammonium Formate in IPA: ACN = 9:1 with 0.1% formic acid) with the following gradient: 85% A with for 3.25 min, then gradually decreased to 18% in 16.2 min, and maintain it for 1 min before setting A at 1% for 0.8 min. Equilibrium was set for 5.6 min from 18.4 to 23 min. The flow rate and column temperature were set to 0.2 mL/min and 50℃, respectively. Full scan and LC/MS/dd-MS modes of UPLC-Orbitrap were used to detect untargeted and targeted metabolites.

For untargeted metabolomics, raw data were processed using MZmine (ver 3.7.2). The final feature list was analyzed using Metaboanalyst 4.0 (*https://www.metaboanalyst.ca/*) and metabolites with a |fold change| > 1.2, and *p*-value < 0.05, were considered to be significantly changed, which were further annotated using MS/MS spectra database of MSDial ver 4.70.

**TLC for TG analysis**

The process of TG analysis by TLC has been described previously (3). An equal volume of tumor (40 mg) was homogenized and incubated twice in 400 µL extraction buffer (hexane: isopropanol = 3:2) for 10 min. Extractions were pooled and dried completely in a vacuum evaporator for 30 min, and then the pellet was dissolved in 60 µL of extraction buffer. Palmitate triglyceride (Cat# A10922, Alfa aesar, Thermo Fisher Scientific) standard was dissolved in the extraction buffer at 50 µg/mL. Then, 5 µL of sample or standard was spotted on a silica gel 60 TLC glass plate (Cat# 105721, Merck, Darmstadt, Germany). The plates were placed in a TLC tank with a mobile phase (hexane: diethyl ether: acetic acid at 80:20:1). After separation, the plates were air dried and dipped into 0.2% Amido Black 10 B (Cat# 195243-25G, Sigma-Aldrich) in 1 M NaCl for 20 min. After staining, the plates were washed with water and then with 1 M NaCl several times, and air-dried overnight.

**Stable isotope-labeled acetyl-CoA**

The cells were treated with 150 µM potassium palmitate (U-^13^C_16_) (Cat# CLM-3943-0.5, Cambridge Isotope Laboratories, Inc.) conjugated to the free fatty acid BSA for 24 h. The cells were washed three times with cold PBS, harvested into the new 2.0 mL EP tubes, and then one-phase extracted using freeze-thaw cycles (three times) in MeOH: ACN: DW (5: 3: 2). The mixture was centrifuged at 20,000 × g at 4℃ for 20 min. The supernatant was collected in new EP tubes and used for MS injection. A BEH amide column was used with buffer A (10 mM Ammonium acetate (Cat# 5.33004.0050, Sigma Aldrich) in DW (pH 9.6)) and buffer B (10 mM ammonium acetate in DW:ACN = 2:8 (pH 9.6)). Acetyl-CoA was detected in SIM mode: precursor ion [M+H]^+^ (m/z): 810 for acetyl-CoA and 812 for ^13^C-acetyl-CoA.

**Single cell RNA analysis**

scRNA-seq data from Zhang et al. (4) were analyzed according to the Seurat pipeline for quality control, clustering, normalization, integration, and visualization provided in Zhang et al.’s paper. We downloaded the entire dataset stored in 3CA (*https://www.weizmann.ac.il/sites/3CA/*) and extracted the single-cell sequencing data of five ccRCC tumor samples with VHL-MT (SI_19703, SI_22368, SI_22604, SI_23459, and SI_23483) and two ccRCC tumor samples with VHL-WT (SI_18854 and SI_18855). For quality control, cells with less than 200 RNA features, a high mitochondrial fraction (> 20%; according to Zhang’s paper, the kidney cells were characterized by a high mitochondrial fraction), less ribosome fraction (< 5%), and log10 genes perUMI < 0.8 were removed. Next, data was normalized and integrated by functions “NormalizeData” and “IntegrateLayers” of Seurat package, respectively. The cells were clustered and visualized in two dimensions via UMAP plots using the rpca method.

**TCGA data analysis**

Gene expression RNA-seq and clinical characteristics of the KIRC TCGA data (*version 2017/10/13*) were downloaded from UCSC Xena (*https://xenabrowser.net/*). The RSEM package was employed to quantify transcription, and outcomes were presented as log2(norm-count+1). The correlation between *CEBPB* and *GPD1L* with other genes in ccRCC was downloaded from cBioportal (TCGA Pancancer data), and RSEM unit was used. Next, the list of genes with Spearman correlation was loaded to Gene Set Enrichment Analysis (GSEA) and run GSEAPreranked with various gene sets databases (Hallmark, Genomic Oncology, KEGG). We applied the cutoff 0.05 for FDR and 0.05 for adjust *p*-value.

**Structure-based virtual screening and docking**

The virtual screening and docking procedure were performed using AutoDock-gpu version 1.5.3 and the associated tools such as AutoDock-tools or MGL-tools obtained from [*https://github.com/ccsb-scripps/AutoDock-GPU*](https://github.com/ccsb-scripps/AutoDock-GPU) (5). The source was compiled with the NVIDIA CUDA toolkit by GCC compiler under Windows Subsystem for Linux 2 environment running in an Intel Core i7 computer system equipped with an NVIDIA RTX3070 GPU. The crystal structure of the CEBPB-DNA complex (PDB ID: 8K8D) was obtained from the protein data bank ([*http://rcsb.org*](http://rcsb.org)) and converted to the pdbqt format using AutoDock tools. Individual map files were generated using autogrid4 program with 40, 47, 82 grid points and 0.102, -0.282, 41.348 coordinates in XYZ axis, respectively, and A, C, NA, OA, N, HD, S, F, and Cl atom types. For ligands, a three-dimensional structural library of ligands was also obtained from the ligand expo site in the protein data bank (*http://ligand-expo.rcsb.org/ld-download.html*). As the downloaded files contain many redundant PDB entities containing a particular ligand, only one unique PDB file was retained for a ligand. Partial charge was added to the individual PDB files using an AutoDock tools script (prepare_ligand4.py), generating the pdbqt files for ligands. A total of 29,314 ligand files was docked against the CEBPB-DNA complex structure for the virtual screening. Results were sorted according to estimated free energy of binding. Among the lowest 10 binding energy results, mithramycin derivatives and mogroside derivatives appear twice and final three compounds (mithramycin, BIO8898, and maytansine) were both commercially available and cell-penetrable. Out of the three, BIO8898 exhibited selective inhibition on ccRCC cells (**Figs. S8 and 8D**).

**Statistical analysis**

Each biological experiment was replicated at least three times (n ≥ 3), consistent with previously published work using similar experimental conditions (6). The results of the replicates are presented as the mean ± SD. Statistical analysis was performed using the GraphPad Prism 10.2.1 software. The un-paired *t*-test was used to analyze two sets of data, while two-way ANOVA with Geisser-Greenhouse correction and a post hoc Dunnett-*t*-test was applied for multiple comparisons. We used the Mann-Whitney U test to establish statistical significance for TCGA non-parametric data.

**References:**

1. Wu J, Yang S. A simulation-based sample size calculation method for pre-clinical tumor xenograft experiments. J Biopharm Stat. 2018;28(3):437-50.

2. Vichai V, Kirtikara K. Sulforhodamine B colorimetric assay for cytotoxicity screening. Nature Protocols. 2006;1(3):1112-6.

3. McLelland GL, Lopez-Osias M, Verzijl CRC, Ellenbroek BD, Oliveira RA, Boon NJ, et al. Identification of an alternative triglyceride biosynthesis pathway. Nature. 2023;621(7977):171-8.

4. Zhang Y, Narayanan SP, Mannan R, Raskind G, Wang X, Vats P, et al. Single-cell analyses of renal cell cancers reveal insights into tumor microenvironment, cell of origin, and therapy response. Proc Natl Acad Sci U S A. 2021;118(24).

5. Santos-Martins D, Solis-Vasquez L, Tillack AF, Sanner MF, Koch A, Forli S. Accelerating AutoDock4 with GPUs and Gradient-Based Local Search. J Chem Theory Comput. 2021;17(2):1060-73.

6. Chen L, Liu Y, Zhang J, Song T, Wu J, Ren Z. AMPK regulates ARF1 localization to membrane contact sites to facilitate fatty acid transfer between lipid droplets and mitochondria. Cell Death Dis. 2025;16(1):623.
