# Supplementary material for: Epigenetically-controlled CEBPB regulates kidney cancer tumorigenesis via GPD1L-mediated ether lipid synthesis
Source: Cell Death Dis. 2026 Jan 22;17(1):175. doi: 10.1038/s41419-025-08403-4 (PMC12877132; doi:10.1038/s41419-025-08403-4)

Figure 2A:

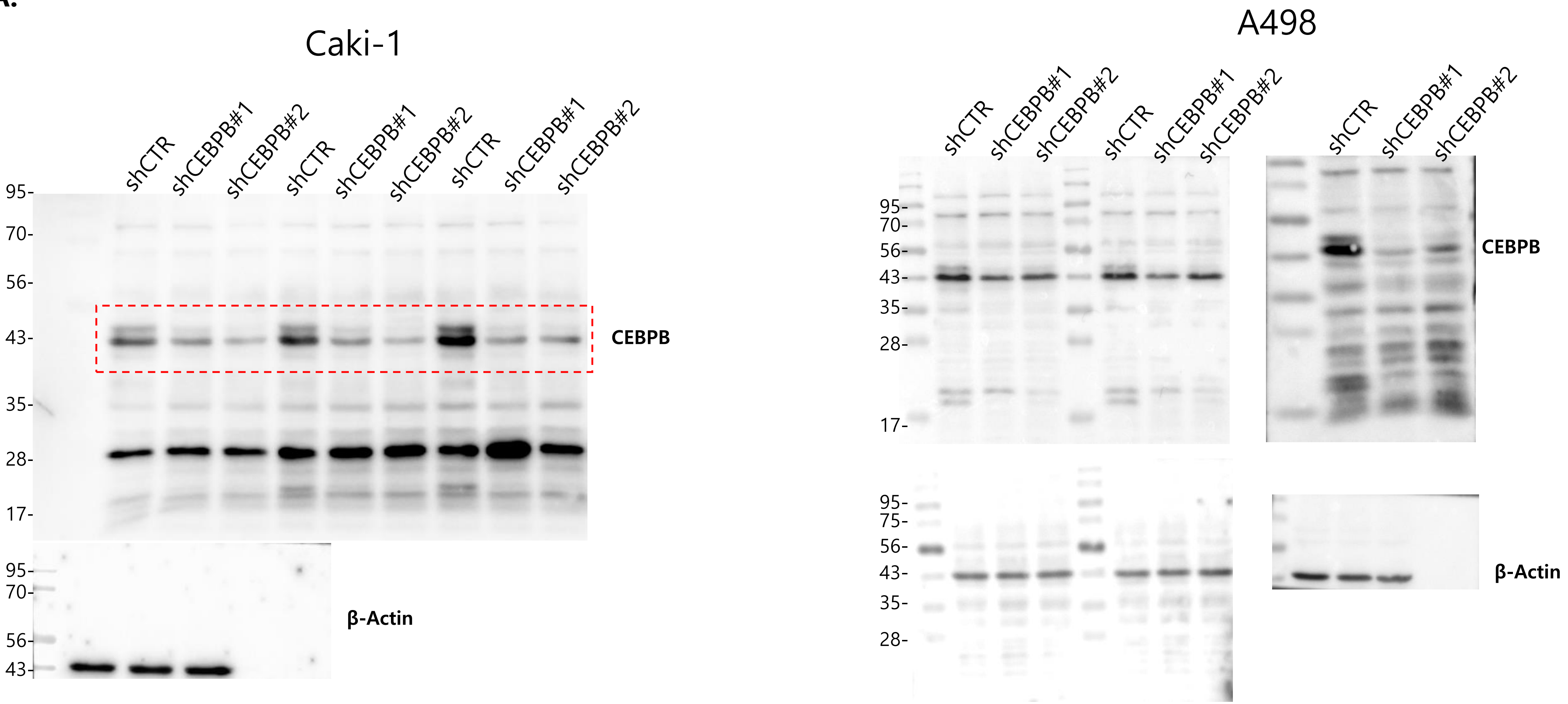
Caki-1
shCTR
shCEBPB#1
shCEBPB#2
shCTR
shCEBPB#1
shCEBPB#2
shCTR
shCEBPB#1
shCEBPB#2
95-
70-
56-
43-
35-
28-
17-
CEBPB
95-
70-
56-
43-
β-Actin
A498
shCTR
shCEBPB#1
shCEBPB#2
shCTR
shCEBPB#1
shCEBPB#2
shCTR
shCEBPB#1
shCEBPB#2
95-
70-
56-
43-
35-
28-
17-
CEBPB
95-
75-
56-
43-
35-
28-
β-Actin

Figure 4A:

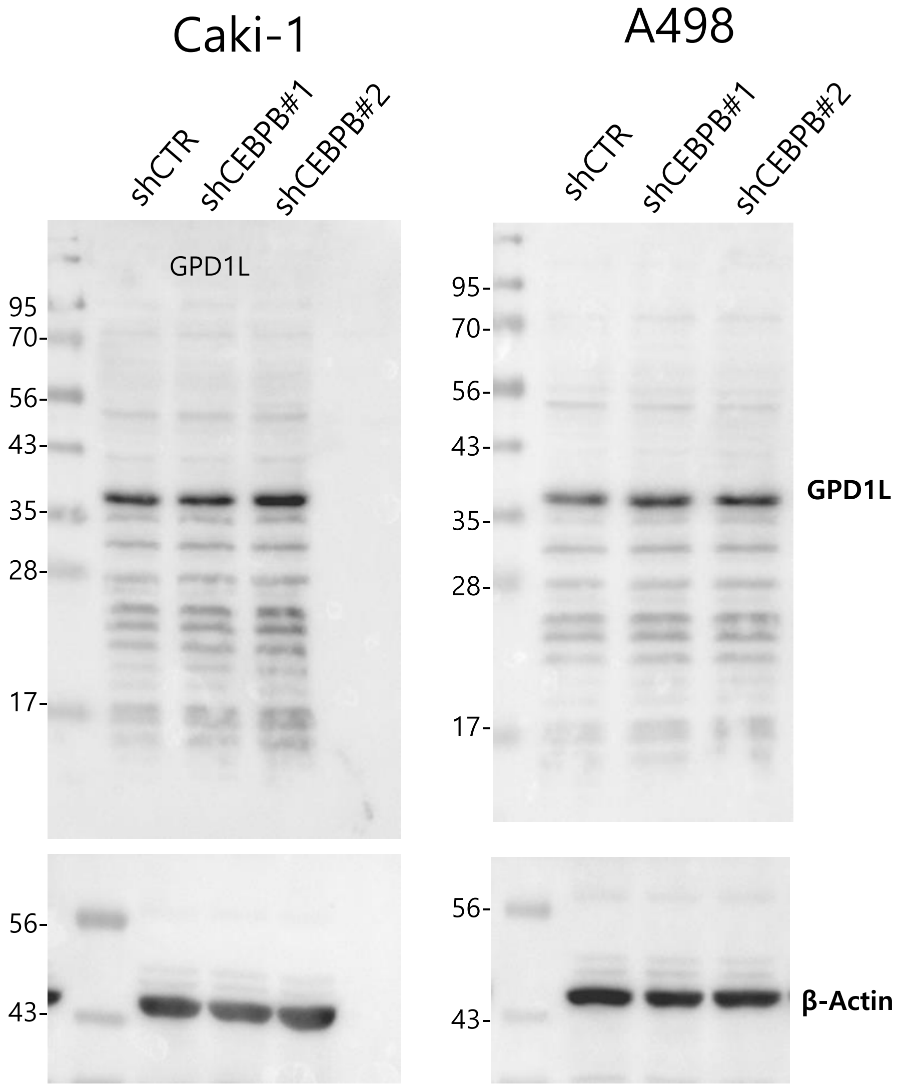

Figure 4G:

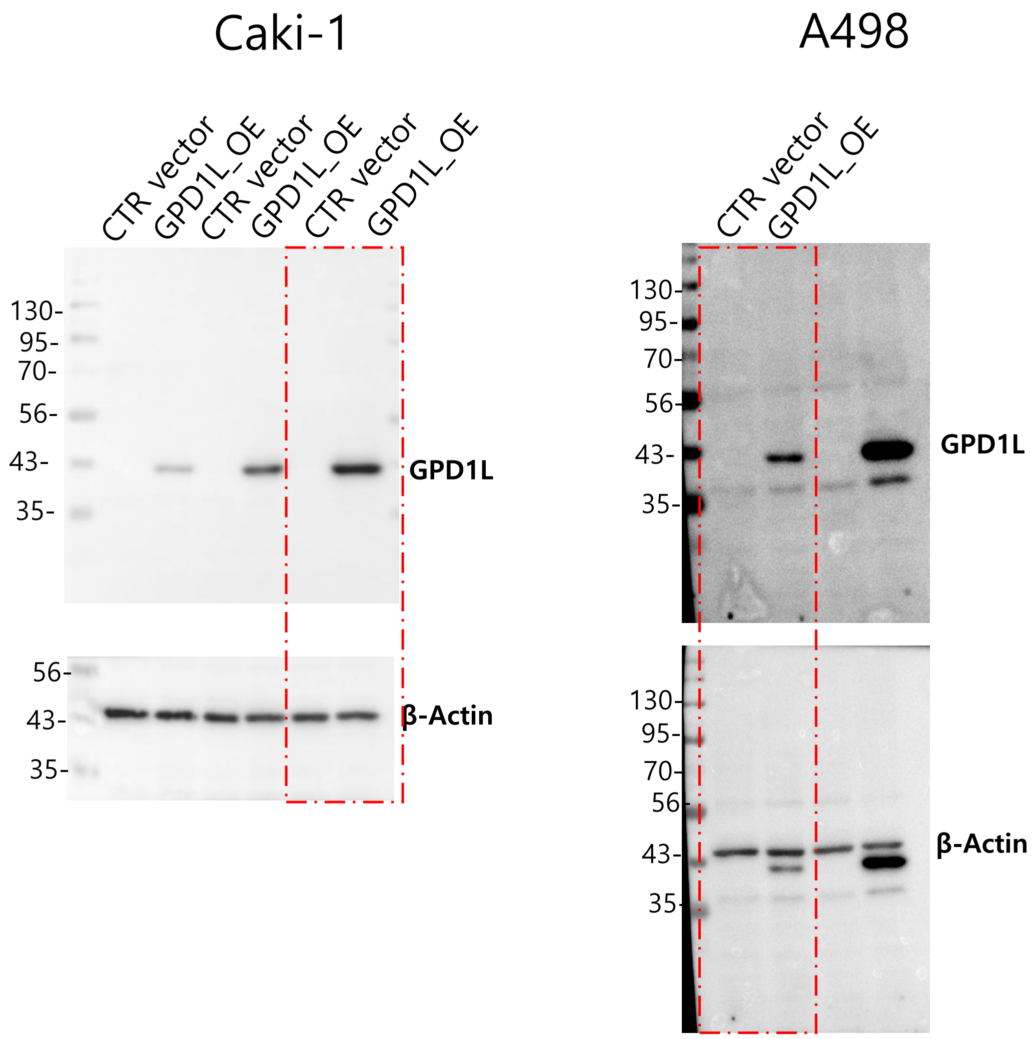

Figure 5A:

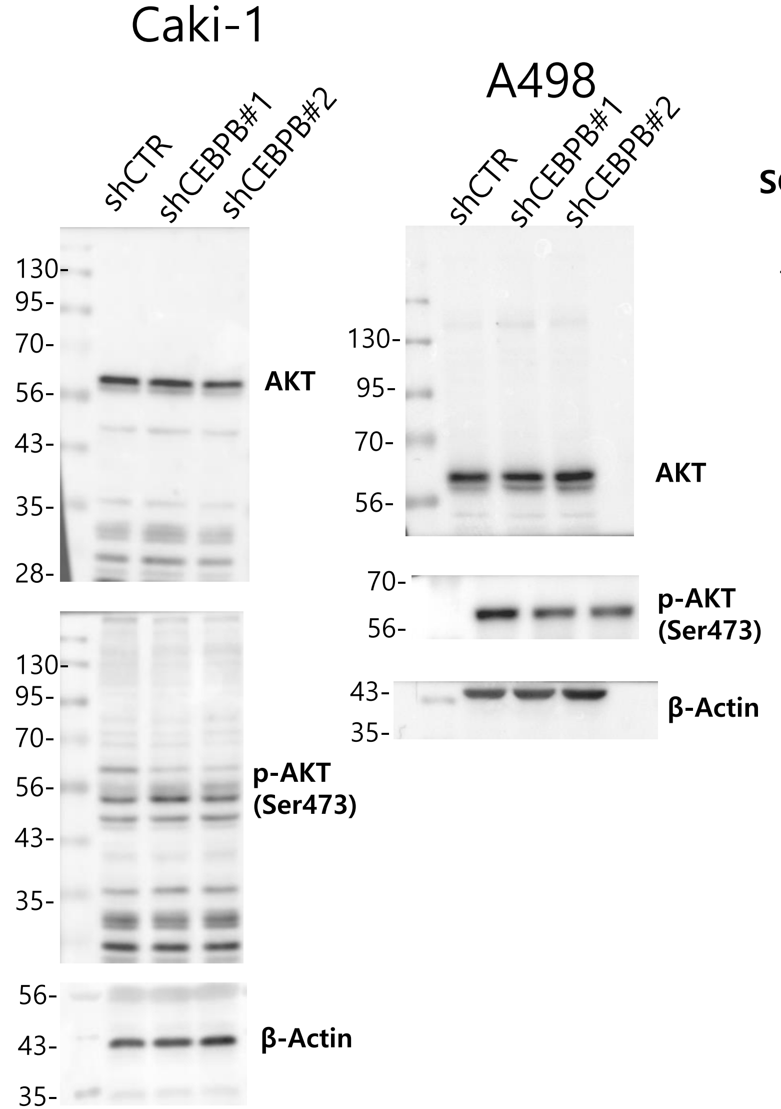

Figure 5B:

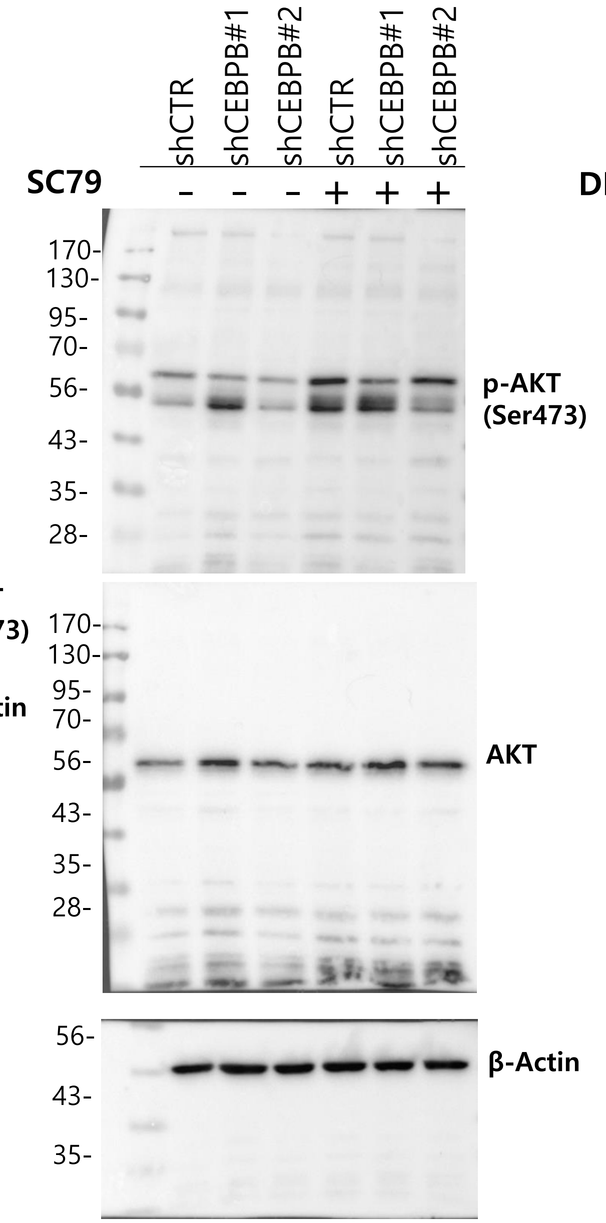

Figure 5D:

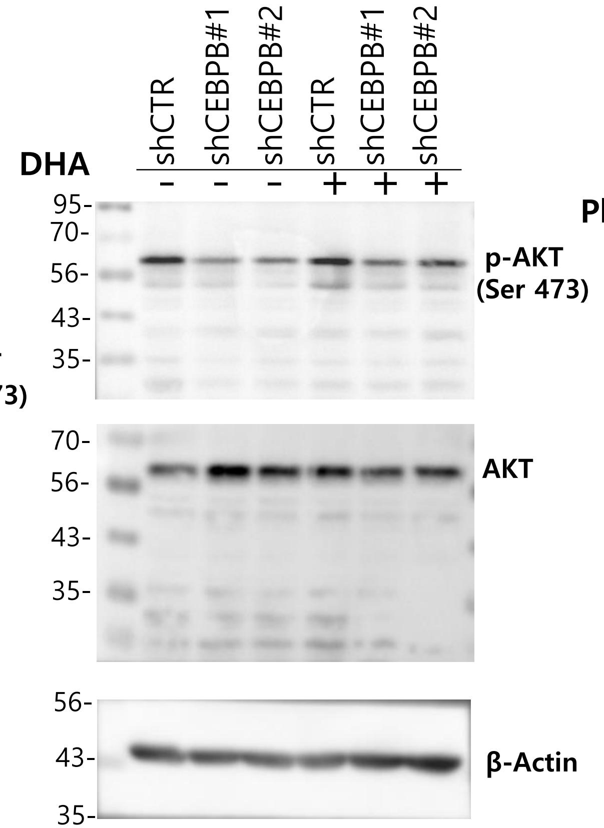

Figure 5E:

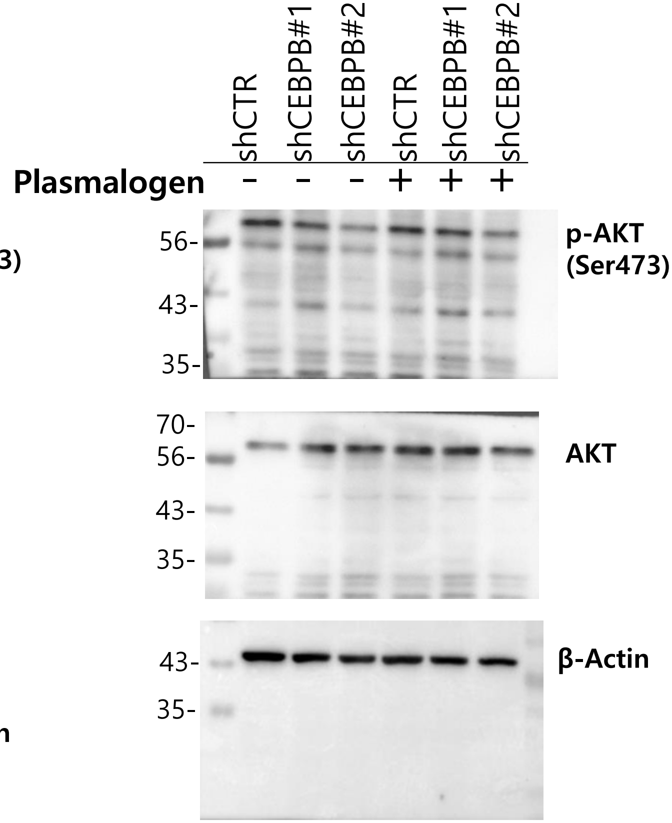

Figure 6C:

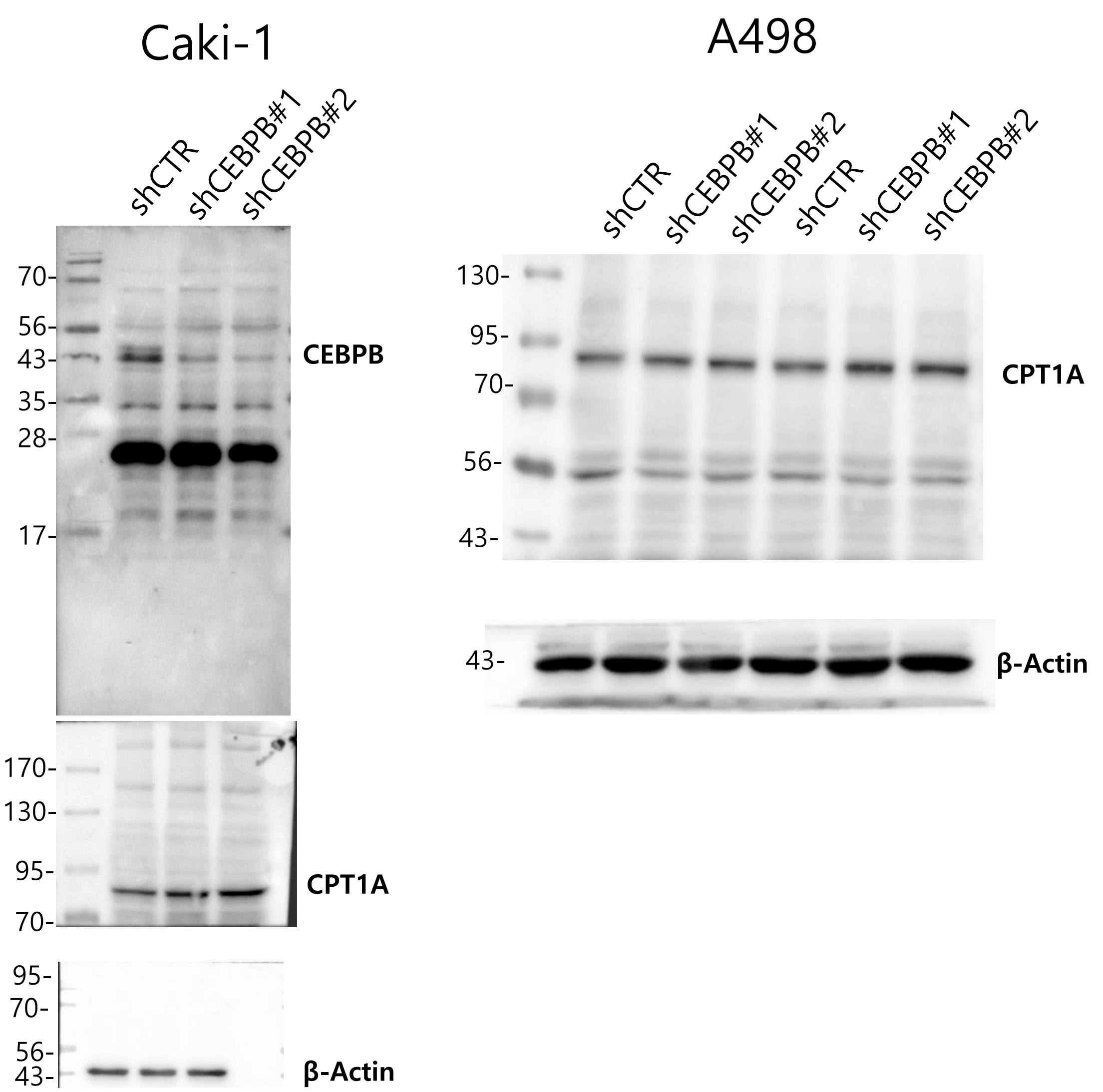

Figure 6D:

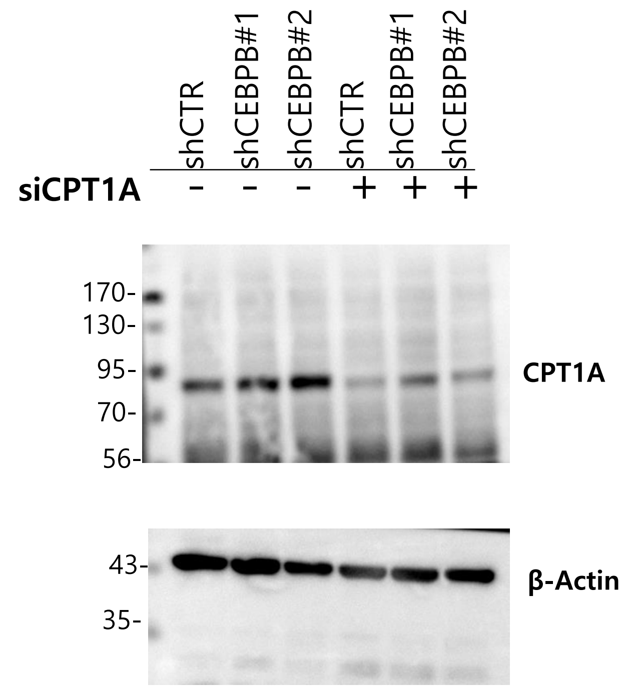

Figure 6H:

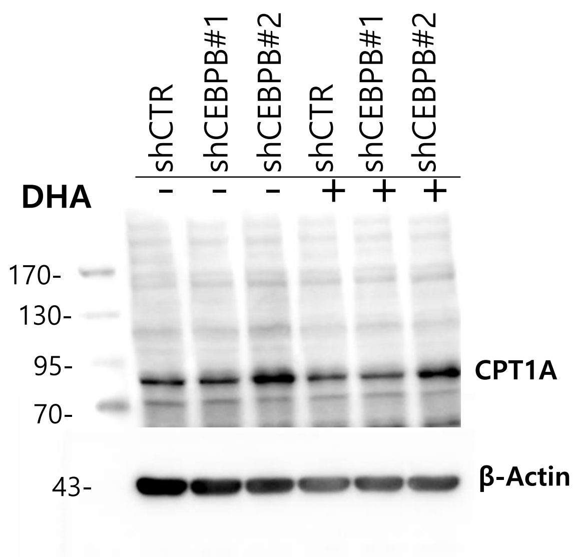

Figure 6I:

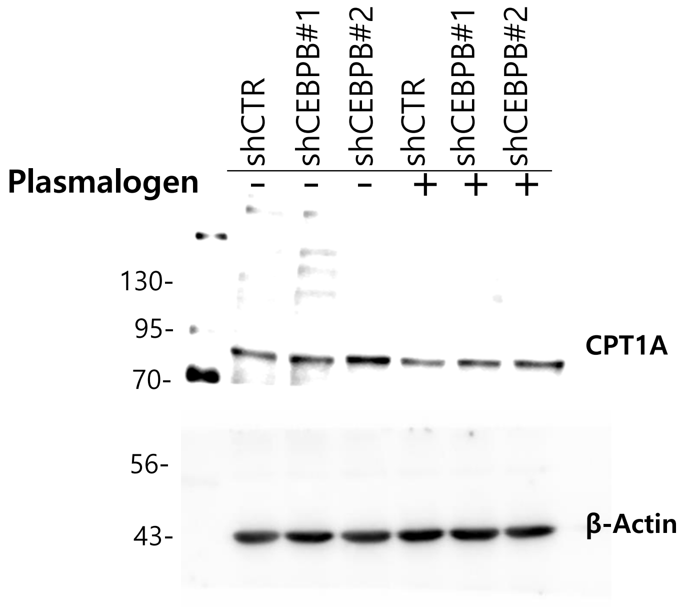

Figure 6J:

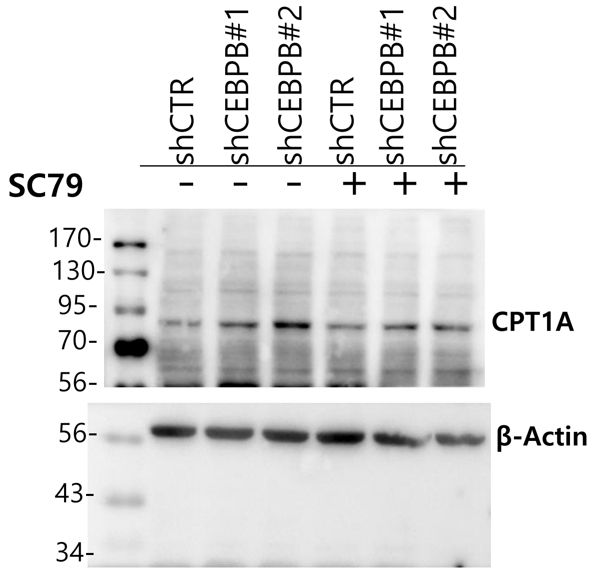

Figure 7E:

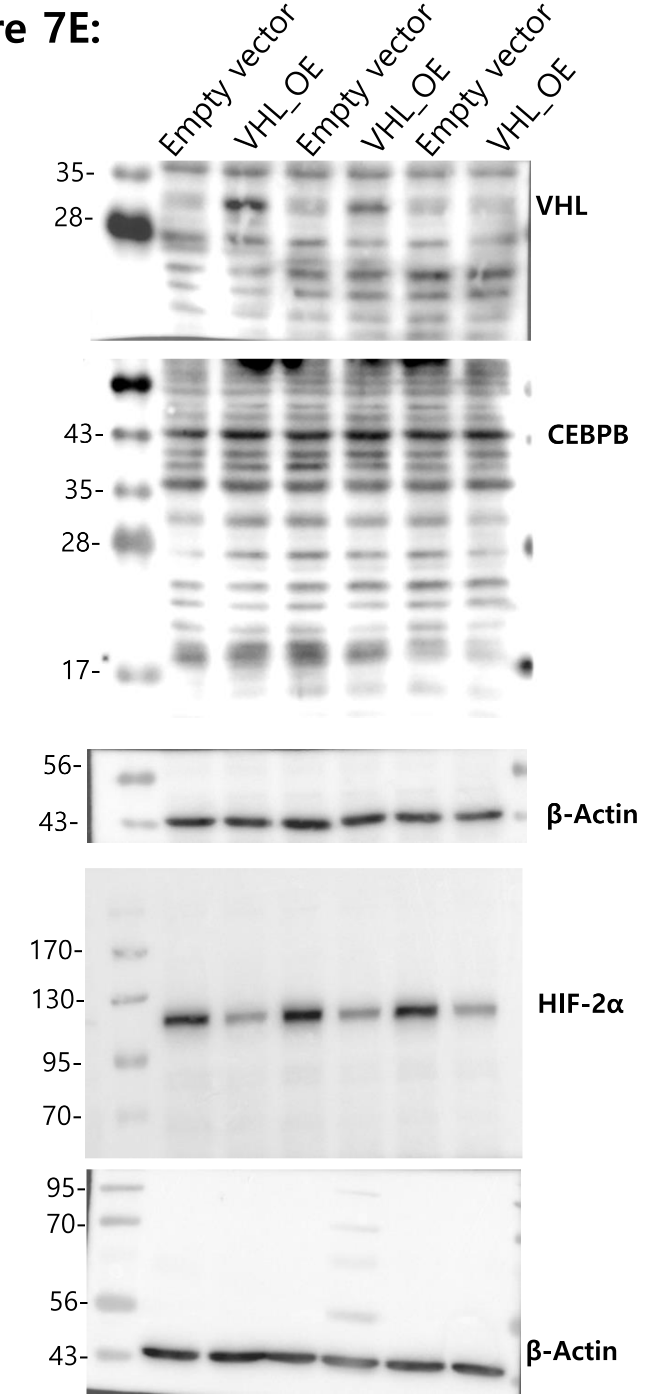

Figure 7F:

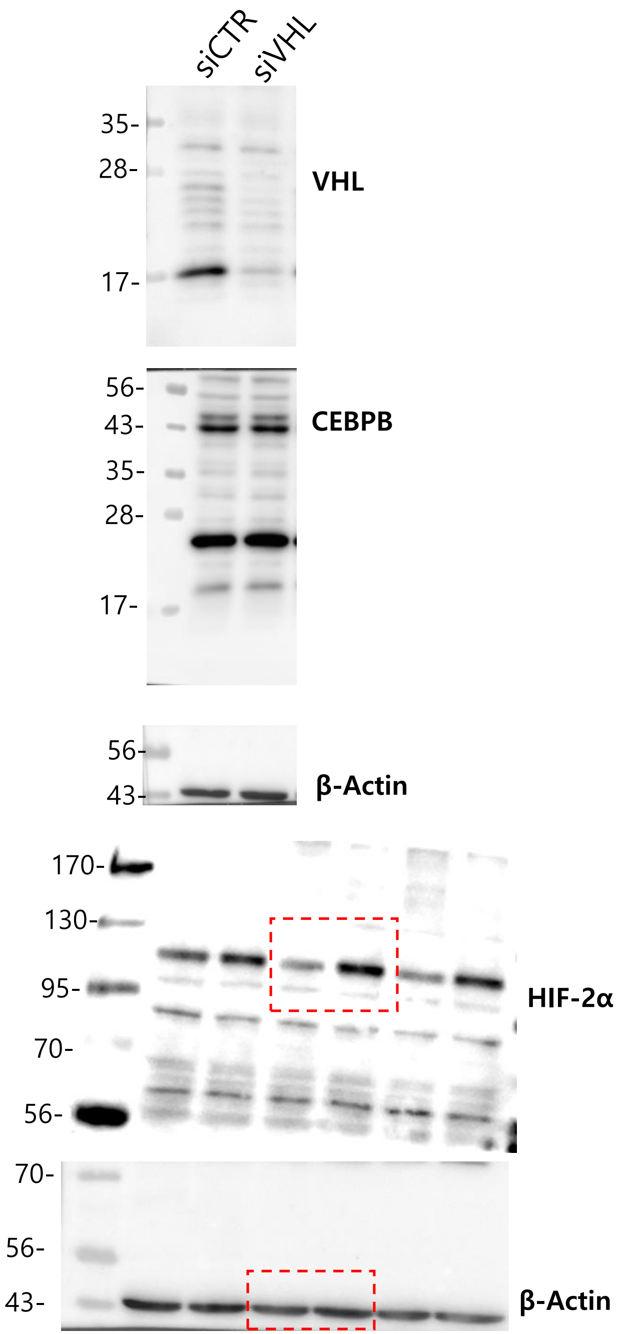

Figure 7G:

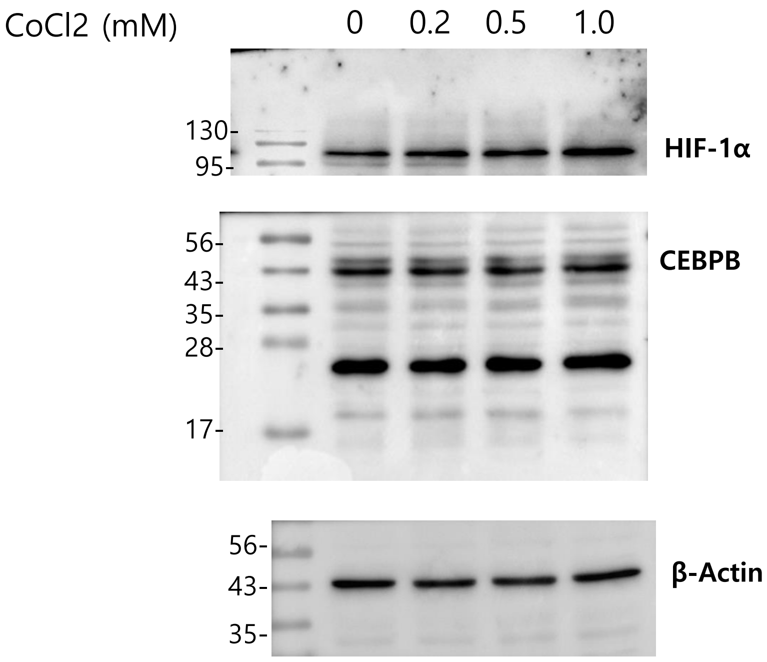

Supplementary Figure S2H:

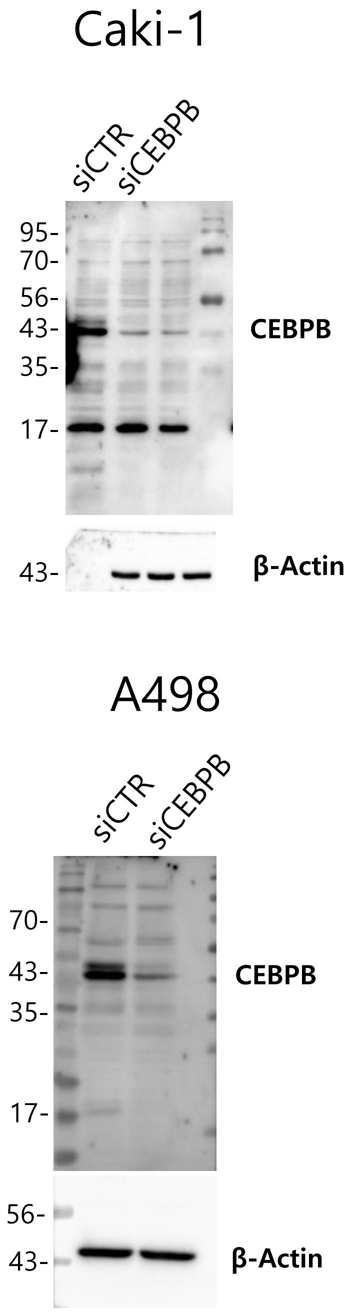

Supplementary Figure S4A:

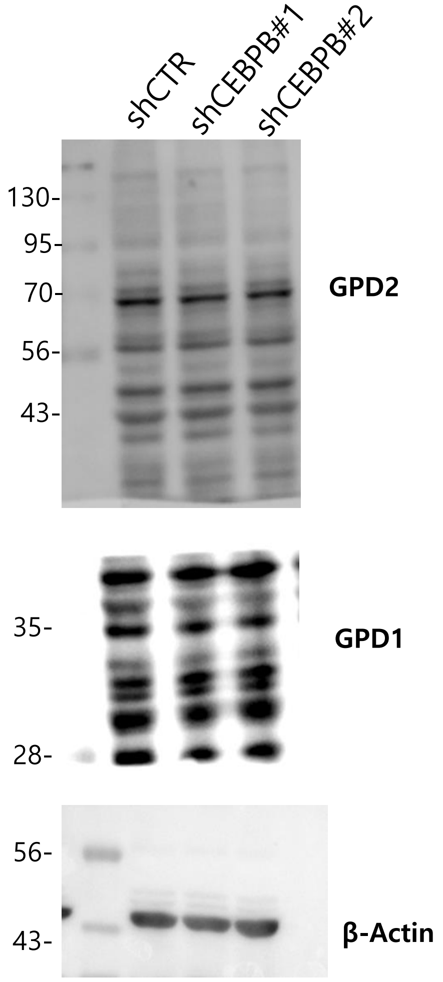

Supplementary Figure S5C:

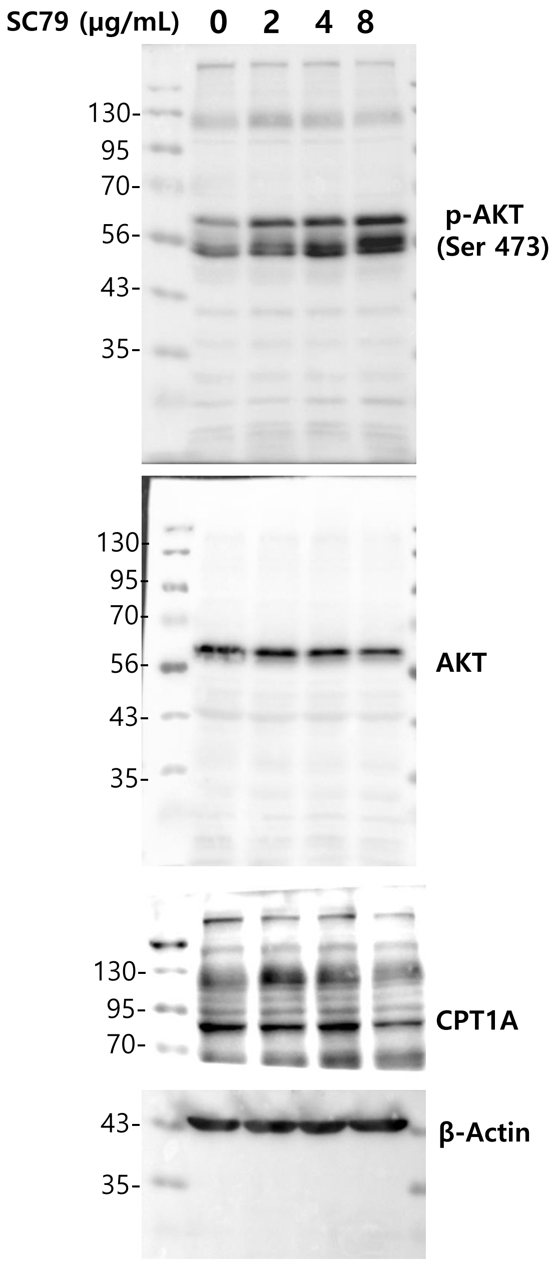

Supplementary Figure S7A:

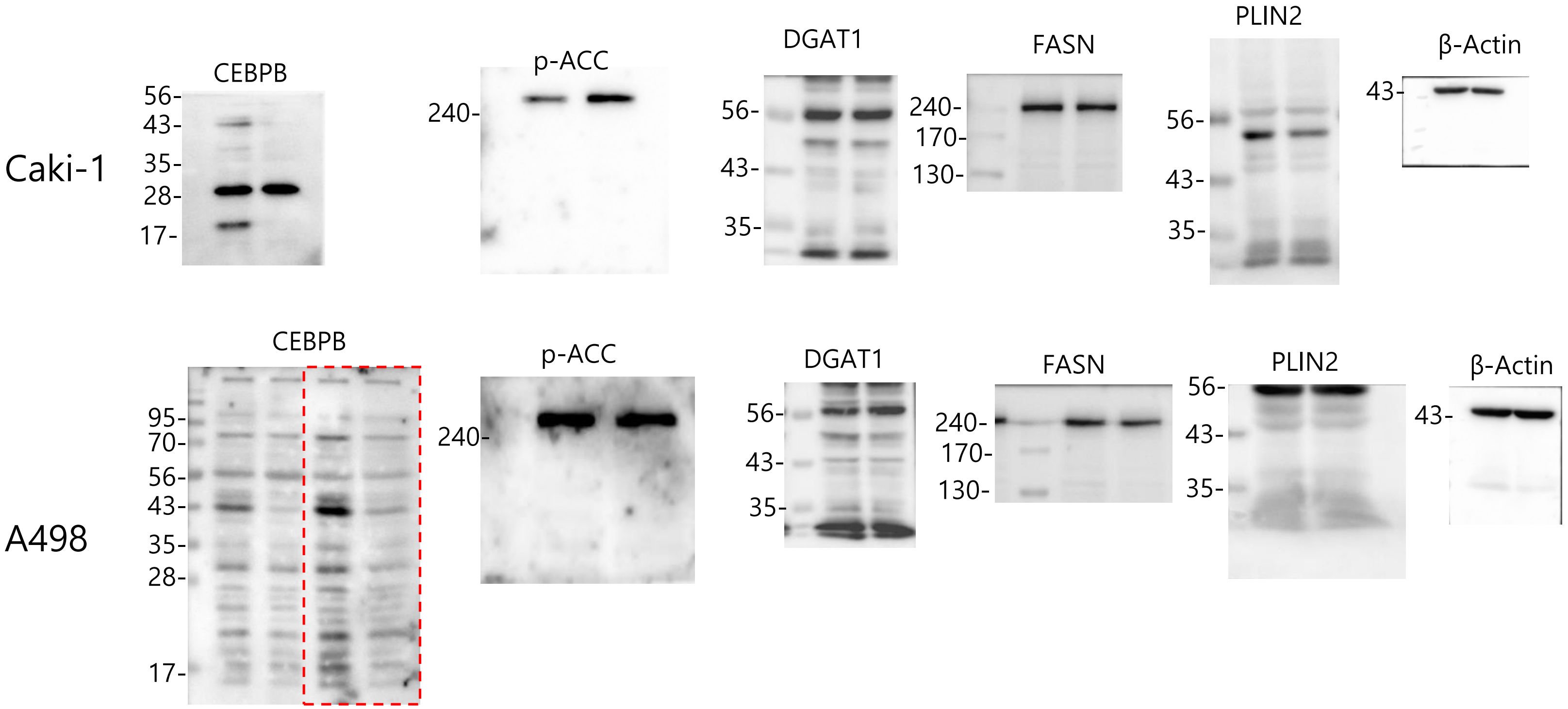

Supplementary Figure S7B:

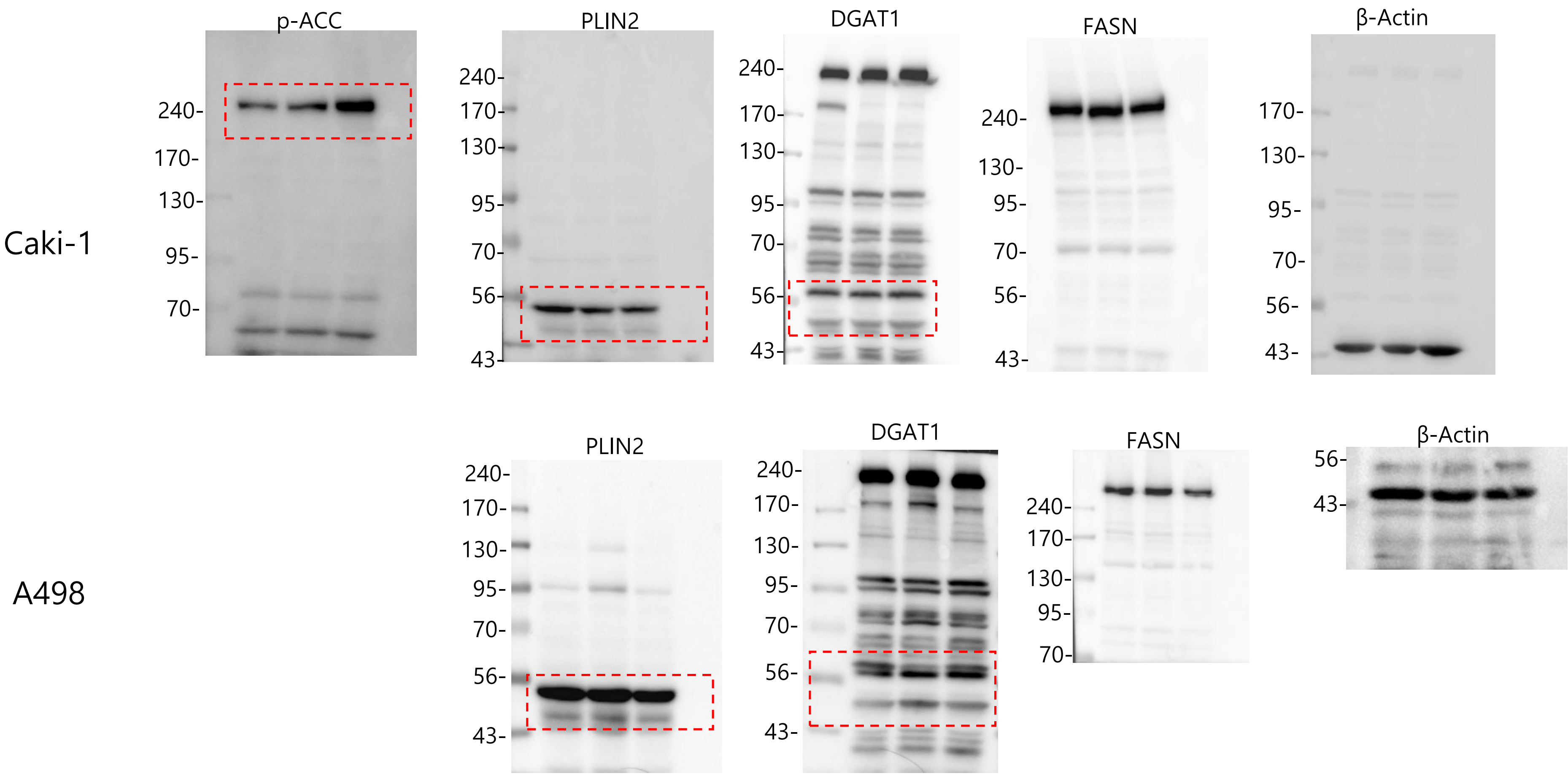

Supplement: Supplementary file 2 — Full and uncropped western blots [file 41419_2025_8403_MOESM2_ESM.pdf]
